# Recent Advances of Electrocatalysts and Electrodes for Direct Formic Acid Fuel Cells: from Nano to Meter Scale Challenges

**DOI:** 10.1007/s40820-025-01648-w

**Published:** 2025-02-17

**Authors:** Yang Li, Ming-Shui Yao, Yanping He, Shangfeng Du

**Affiliations:** 1 School of Chemical Engineering, University of Birmingham, Birmingham, B15 2TT UK; 2 Department of Chemical Engineering, Loughborough University, Loughborough, Leicestershire LE11 3TU UK; 3 State Key Laboratory of Mesoscience and Engineering, Institute of Process Engineering, Chinese Academy of Sciences, Beijing, 100190 People’s Republic of China; 4 University of the Chinese Academy of Sciences, Beijing, 100049 People’s Republic of China; 5 School of Chemical Engineering, Kunming University of Science and Technology, Kunming, 650504 People’s Republic of China

**Keywords:** Direct formic acid fuel cell, Electrocatalyst, Electrode, Formic acid oxidation, Mass transfer

## Abstract

Direct formic acid fuel cells are promising energy devices with advantages of low working temperature and high safety in fuel storage and transport. They have been expected to be a future power source for portable electronic devices. The technology has been developed rapidly to overcome the high cost and low power performance that hinder its practical application, which mainly originated from the slow reaction kinetics of the formic acid oxidation and complex mass transfer within the fuel cell electrodes. Here, we provide a comprehensive review of the progress around this technology, in particular for addressing multiscale challenges from catalytic mechanism understanding at the atomic scale, to catalyst design at the nanoscale, electrode structure at the micro scale and design at the millimeter scale, and finally to device fabrication at the meter scale. The gap between the highly active electrocatalysts and the poor electrode performance in practical devices is highlighted. Finally, perspectives and opportunities are proposed to potentially bridge this gap for further development of this technology.

## Highlights

- Comprehensive review of the progress in direct formic acid fuel cells from catalytic mechanisms to catalyst design, and to the electrode/device fabrication.
- The gap between highly active formic acid oxidation catalysts and unsatisfactory device performance is highlighted.
- Perspectives for catalyst and electrode design are discussed.

## Graphical abstract

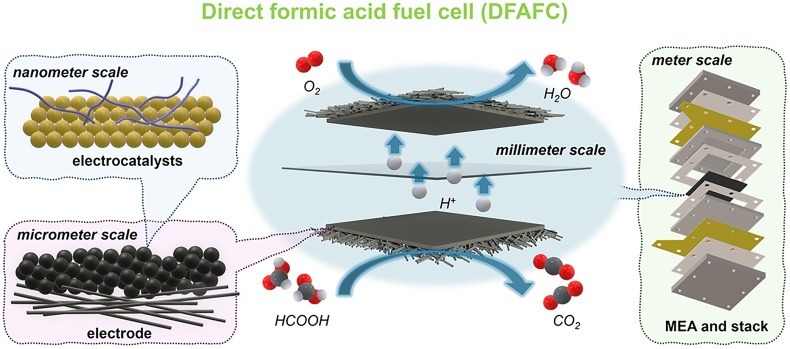

## Introduction

**Fig. 1:**
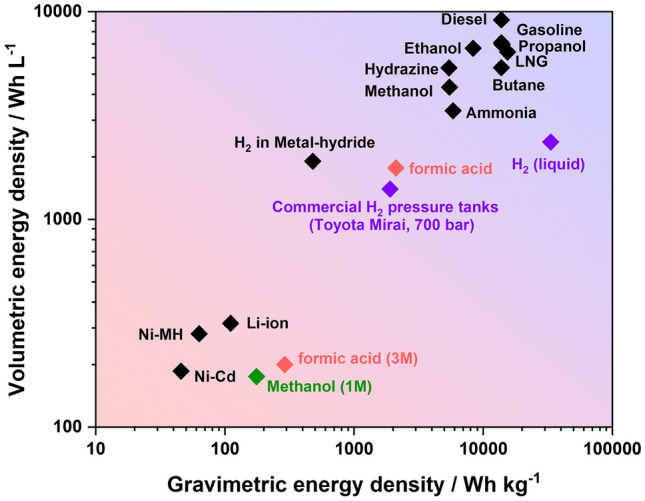
Gravimetric and volumetric energy density of different fuels

Over the last decades, the pace of research and development of clean and sustainable energy technologies has sharply increased, motivated by the growing energy demand and pressures of environmental challenges. Proton exchange membrane fuel cells (PEMFC), as one of the clean power generation technologies, have become a crucial industrial sector for global sustainable economic development [1]. In the history of the PEMFC, most of the efforts were spent on hydrogen-PEMFC. By contrast, these intensive studies can still not solve the inherent limitation of hydrogen, in particular, the challenges facing hydrogen storage and distribution. Driven by this limitation and the requirements of alternative clean power sources, liquid fuels, including methanol, ethanol, formic acid, ammonia, etc., have received more attention in the fuel cell area. Most of these fuels are considered as safe and convenient for storage and operation, and can be obtained either through sustainable approaches, or by catalytic reforming of abundant fossil fuels such as natural gas. Besides, most liquid fuels have a competitive energy density compared with high-pressure or even liquid hydrogen (shown in Fig. 1), which can even be several orders of magnitude larger than that of the Li-ion battery [2].

The history of the first liquid fuel cell can be traced back to 1845 [3], six years after the first fuel cell (in the former time, it was known as “gas battery”) demonstrated by Sir William Grove [4], in which he used gas battery to ascertain voltaic relation of oxygen and alcohol. In light of the advancements in hydrocarbon fuel infrastructure, in the early studies on liquid fuel cells, researchers had several attempts on hydrocarbons, such as using diesel or jet fuel as the power sources [5]. However, these studies showed less positive results due to the large challenges in the electrooxidation of hydrocarbons at both low- and intermediate-temperature. This limitation later made the research focus shift to direct methanol fuel cells (DMFC). Since the last century, huge efforts have been carried out on DMFC research [6–10]. The fast development of advanced electrochemical equipment provided opportunities to investigate the mechanism of the methanol oxidation reaction (MOR); thus, a great number of studies were conducted in order to increase the power performance of DMFC [11–17]. Driven by the great development of DMFC at that period, the idea of the “Methanol Economy” was proposed as an alternative energy source to the “Hydrogen Economy” [18].

While the studies on the DMFC had been developed for a long time, many challenges remained unaddressed, in particular, catalyst poisoning and fuel crossover [19]. Therefore, scientists began to explore alternative liquid fuels. Formic acid is a safe liquid on the list of food additives published by the US Food and Drug Administration (FDA). The thermodynamic potential (*E*_*0*_) of formic acid oxidation (FAOR) is -0.25 V vs RHE, compared to the hydrogen oxidation (0 V vs RHE) and MOR (+ 0.03 V vs RHE). Benefiting from this, the theoretical open-circuit voltage (OCV) of the direct formic acid fuel cell (DFAFC) can reach 1.48 V, higher than 1.23 V of hydrogen-PEMFC and 1.20 V of DMFC [20, 21]. Also, during the DFAFC operation, the repulsion between the HCOO^−^ group of formic acid and the sulfuric group in the commonly used perfluorinated sulfonic acid (PFSA) membrane leads to a smaller crossover flux, which offers an opportunity for using a high concentration of formic acid to provide a high power density [1, 22]. Moreover, as an important component of carbon neutrality, formic acid can be directly produced by carbon dioxide reduction. If CO_2_ generated from FAOR is used, a CO_2_ loop with a net zero can be achieved. These advantages make DFAFC a highly competitive power source for future applications.

Rapid research and development of DFAFC contributed to many improvements in the most recent years, and demonstrated better commercialization prospects [23]. In order to better understand the recent fast progress on both FAOR catalysts and electrodes for DFAFC, a new review paper based on the state-of-the-art studies is required to summarize the progress achieved and give a clear picture of the current strategies and methods to design and fabricate DFAFC, despite several papers focusing on the specific/narrow aspect have been published previously where usually FAOR electrocatalysts are discussed [24–27]. This article will comprehensively review and analyze the development in the field of DFAFC, including the fundamental principles and challenges of the FAOR, preparation methods of electrocatalysts, strategies for the improvement of catalytic activities and stability, and especially the development of DFAFC electrodes. In particular, the evaluation of these catalysts and the large gap between their intrinsic activities and practical power performance in fuel cell electrodes are highlighted. Finally, the technical challenges are summarized and analyzed with several proposed future research directions to overcome the challenges toward DFAFC and their commercial applications.

## Hierarchical Structures and History of DFAFC

**Fig. 2:**
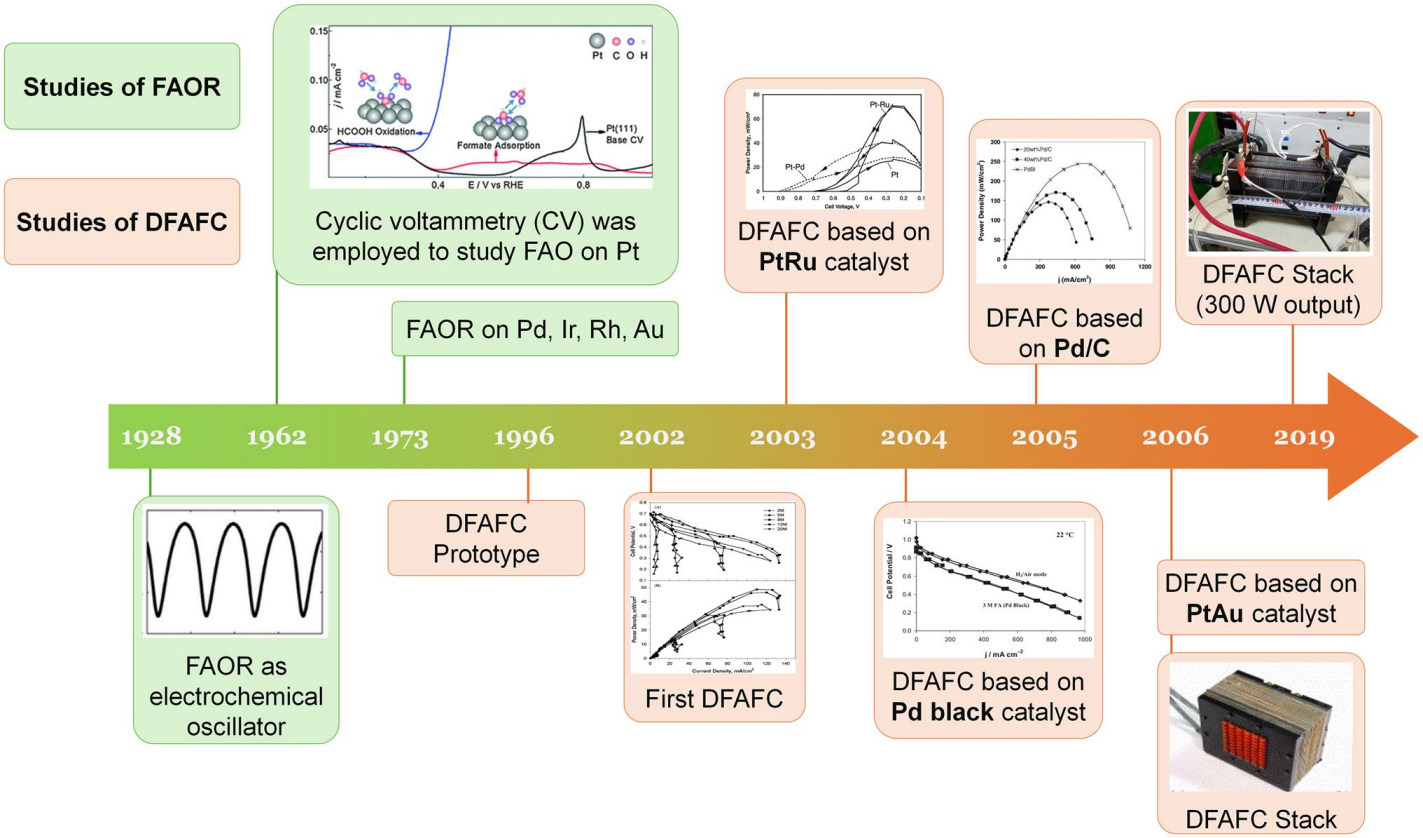
The timeline for the study of the formic acid oxidation reaction (FAOR) and direct formic acid fuel cell (DFAFC). References: **CV** [11, 29], **FAOR on Pd, Ir, Rh and Au** [32, 37], **Prototype** [30], **First DFAFC** [31], **PtRu** [33], **Pd black** [38], **Pd/C** [35], **Stack** [39], **300 W Stack** [23]

Although it has been nearly two hundred years since the concept of the fuel cell was proposed, DFAFC, as a new-born technique in the fuel cell family, was not presented until the end of the last century. The earliest research mainly focused on the mechanism of FAOR due to its simple two-electron exchange during oxidation. As shown in Fig. 2, the investigation of the FAOR can be traced back to 1928 as the best-known example of a nonlinear electrochemical oscillator [28]. With more advanced technology, the mechanism of FAOR was investigated later by using the voltammetry and chronopotentiometry study [11, 29]. Despite researchers putting intensive research efforts and establishing a deep understanding of FAOR, formic acid did not receive attention as the fuel used in fuel cells until 1996 [30], when Weber and co-workers proposed a novel fuel cell prototype based on the principle of FAOR. They found the electrochemical oxidation activity of formic acid was better than methanol on both Pt black and Pt/Ru catalysts under the same conditions. In 2002, the first DFAFC was demonstrated by Rice and co-works [31]. The MEA was made by 7 mg cm^−2^ Pt black (cathode), Nafion® 117 and 4 mg cm^−2^ Pt-based proprietary catalyst (anode), delivering the peak power density of 5 mW cm^−2^ (2 M formic acid) and 48.8 mW cm^−2^ (12 M). In this study, the working temperature was significantly reduced to 60 °C, and this condition is still being used in studies today. After that, benefiting from the long-term research and understanding achieved on FAOR, especially the reaction on different precious metals [32], the development of DFAFC was boomed. Most techniques used now have been proposed in the first few years after the birth of DFAFC, including PtAu- and Pd-based catalysts [33, 34], especially for the practice of carbon-supported catalysts, which significantly reduced the catalyst loading by optimizing the catalyst utilization within fuel cell electrodes [35, 36]. However, due to the lack of a breakthrough in the study of FAOR and DFAFC, the pace of DFAFC development slowed down later. Especially in recent years, only a few published papers reported in the MEA fabrication and single-cell tests, while most studies shifted the pure material research focusing on the synthesis of electrocatalysts toward FAOR and improving their intrinsic activity.

**Fig. 3:**
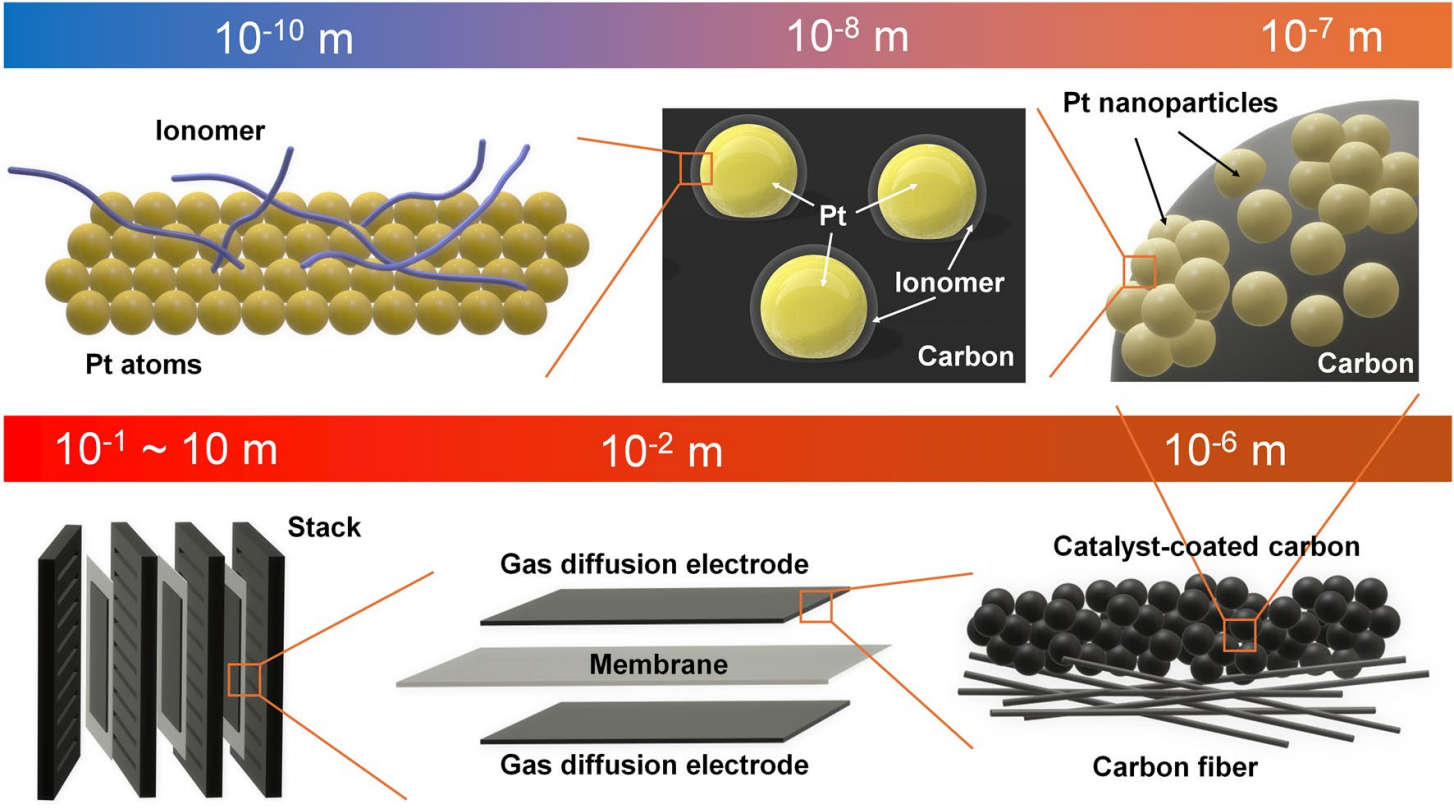
Multilength-scale challenges for DFAFCs

Similar to hydrogen-PEMFC, the study of DFAFC involves a multilength-scale project and requires contributions from different disciplines [40]. As shown Fig. 3, the studies of the smallest scale can be down to atomic size, in which the investigation is carried out to reveal the mechanisms of FAOR on catalyst atoms. With the help of advanced operando techniques and high-performance computing, recent research in this scale primarily focuses on identifying active reaction intermediates, thereby enabling continuous refinement of the FAOR reaction steps. More details can be found below in Sect. 3. Based on the studies of the reaction mechanism, various strategies have been proposed to optimize electrocatalysts, such as increasing the active surface area, enhancing reaction kinetics and avoiding unwanted side reactions. In addition, corresponding synthesis methods are continuously being developed, aiming for cost-effective and environmentally friendly approaches that meet these optimization strategies. These will be detailed in Sect. 4.

Once the synthesized electrocatalyst demonstrated good potential in the electrochemical measurements, further experiments could be designed to fabricate electrodes and membrane electrode assemblies (MEA). At this scale, numerous technical challenges arise, with a key focus on effectively preparing the catalyst to an electrode. This includes methods for spraying the catalyst uniformly, optimizing triple phase boundaries, reducing contact resistance and more. Given that DFAFC research is still in its early stages, studies addressing these areas are limited. This review draws on research from other fuel cells to provide a framework and insights into these aspects. Single-cell and stack tests are necessary in this stage, in which more factors need to be considered, and they will be discussed in Sect. 5.

## Atomic Scale: Formic Acid Oxidation

FAOR is the foundation of catalyst design for boosting their catalytic activities and stability to be used in DFAFC. Based on advanced computing and operando characterization technology, modern research is mainly interested in the identity of the intermediates during the FAOR. This is not only because some of them could poison the catalyst surface, but also because the bond strength of these intermediates is a crucial factor for determining reaction kinetics. This section, therefore, will center on the intermediates during FAOR, and discuss the most accepted mechanisms and theories.

### Mechanism of the Formic Acid Oxidation

As discussed in the above section, due to the simple two-electron exchange, this reaction has been used as a model to explore the oxidation processes of more complex organic fuels (such as methanol), and therefore has been extensively investigated since decades ago. As early as 1928, the experiment was conducted toward FAOR on the platinum surface. The formation of adsorbed poisoning species has been proposed based on the oscillatory behavior of the reaction [28]. A later investigation using IR spectroscopy revealed that this catalytic poison is carbon monoxide (CO) [41]. In 1973, the theory of FAOR through dual parallel pathways was first proposed by Capon and Parsons [42]. With one pathway, formic acid is directly oxidized to carbon dioxide via a dehydrogenation reaction without breaking CO bonds (direct pathway): ${\varvec{H}}{\varvec{C}}{\varvec{O}}{\varvec{O}}{\varvec{H}}\to {{\varvec{C}}{\varvec{O}}}_{2}+2{{\varvec{H}}}^{+}+2{{\varvec{e}}}^{-}$. In another pathway, FAOR occurs via a dehydration reaction and forms adsorbed species as the intermediate (indirect pathway): ${\varvec{H}}{\varvec{C}}{\varvec{O}}{\varvec{O}}{\varvec{H}}\to {-{\varvec{C}}{\varvec{O}}}_{{\varvec{a}}{\varvec{d}}{\varvec{s}}}+{{\varvec{H}}}_{2}{\varvec{O}}\to {{\varvec{C}}{\varvec{O}}}_{2}+2{{\varvec{H}}}^{+}+2{{\varvec{e}}}^{-}$. Adsorbed intermediates formed followed the indirect pathway are easy to be adsorbed on the catalyst surface, blocking active sites and suppressing catalytic activities (catalyst poisoning). Therefore, a great effort of the recent catalyst design focused on improving the catalyst performance by adjusting the ratio between direct and indirect oxidation. For example, it is found that FAOR is very sensitive to different crystal facets, suggesting the reaction rates of these two pathways significantly rely on the surface structure of the electrocatalysts. An early study was conducted to investigate the oxidation kinetics of small organic molecules by cyclic voltammetry (CV) scan, including formic acid, methanol and formaldehyde, on platinum catalysts. They found Pt (100) plane was fully blocked by the intermediates, while the least adsorption was on Pt (111) surface [43].

**Fig. 4:**
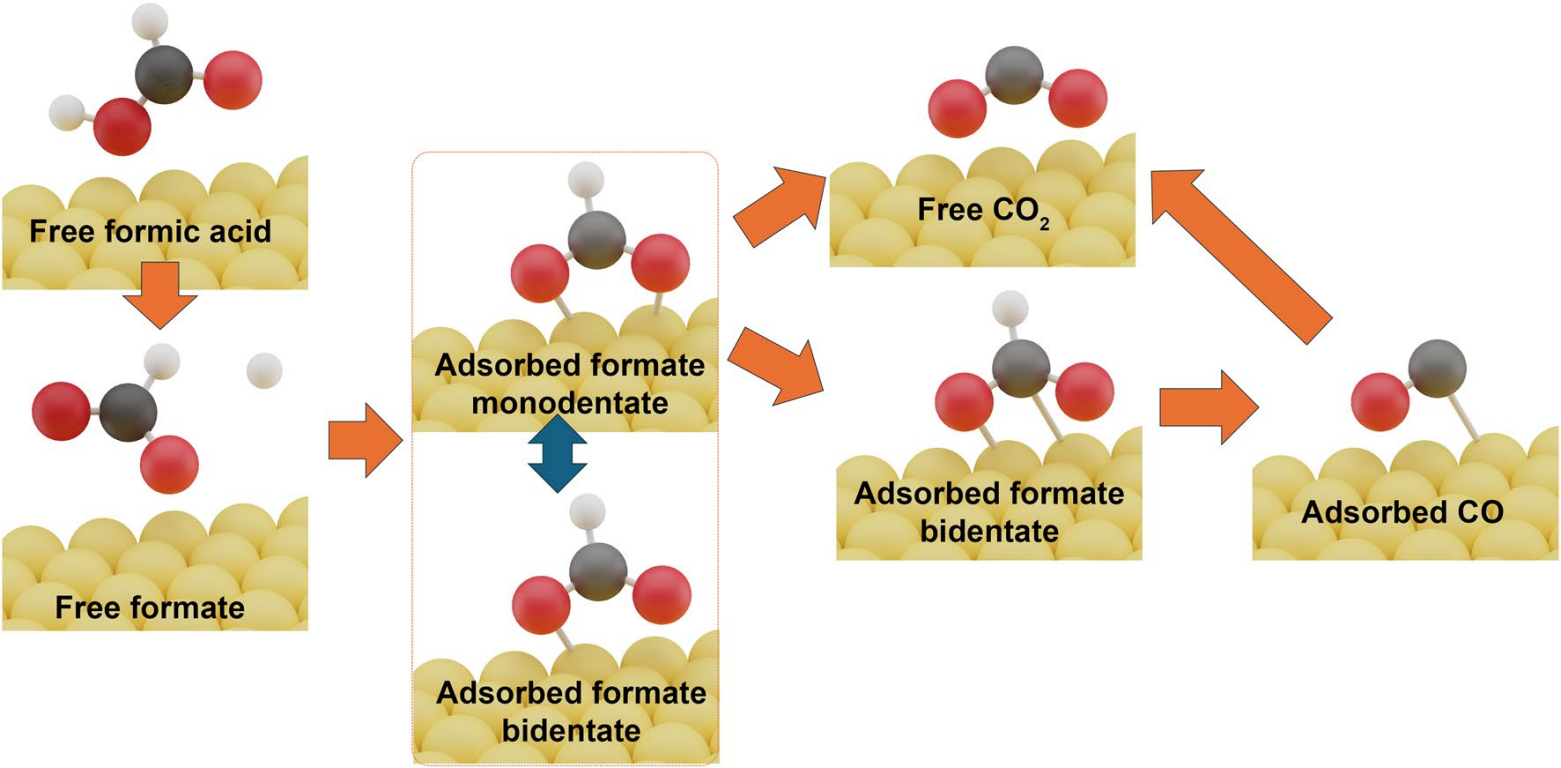
Illustration of the bridge-bonded adsorbed formate mechanism for the formic acid oxidation reaction (FAOR)

However, there is still a lack of complete research to fully describe the mechanism of FAOR, which makes this topic still under debate. The focus of the recent investigation is on the identification of active intermediates. Using advanced surface-enhanced infrared spectroscopy (SEIRAS), adsorbed formate (HCOO_ads_) was initially detected in the direct oxidation pathway [44, 45]. Subsequent studies further indicated this intermediate is also present in the dehydration of formic acid (indirect oxidation) [46, 47]. Moreover, the oxidation of formic acid/formate has been found to exhibit a volcano-shaped pH dependence, peaking at a pH close to the *pKa* of HCOOH (3.75), which suggests that HCOO^−^ oxidation is the dominant reaction pathway across the whole pH range. It is thus posited that formic acid undergoes dissociation into HCOO^−^ prior to oxidation, through acid–base equilibrium [48, 49]. This series of studies is collectively termed the bridge-bonded adsorbed formate mechanism, as shown in Fig. 4.

Given that adsorbed formate is a common active intermediate in FAOR, its adsorption configuration and coverage have received significant attention. Formate has been observed to adsorb on the platinum surface in two configurations: monodentate formate and bidentate formate, depending on the number of oxygen atoms bonded to the platinum. Experimental results suggest that monodentate formate serves as the active intermediate, while bidentate formate is relatively stable [50]. Although bidentate formate does not participate directly in the reaction, it is more than a mere spectator. At low to moderate coverage levels, the presence of adjacent bidentate formate helps stabilize the reactive monodentate formate species [51]. However, when formate coverage exceeds approximately 50%, the rate of direct oxidation decreases, possibly due to the tight packing of bidentate formate, which prevents its conversion to monodentate formate. Additionally, formate could adsorb in another bidentate form, transitioning from a Pt–O adsorption mode to a Pt–C mode, and undergoes a dehydrogenation process to form adsorbed CO [50]. Because the potential of zero free charge (pzfc) on Pt (100) is more negative, high coverage of adsorbed formate can be achieved at relatively negative potentials, resulting in high formate reduction rates and increased CO poisoning. In contrast, on Pt (111), high formate coverage is only achievable at relatively positive potentials, where the reduction of adsorbed formate to CO occurs slowly, explaining the slow CO poisoning observed on Pt (111) electrodes.

However, some studies suggest that formic acid can adsorb directly onto the electrocatalyst surface and undergo oxidation without forming formate as an intermediate [52, 53]. Contrary to previous conclusions, formate may occupy active sites, competing with formic acid and thus poisoning the catalyst. Therefore, catalyst design should selectively prevent formate adsorption. This highlights that our current understanding of the formic acid oxidation is still evolving, necessitating further and more comprehensive research in the future.

Very recently, facilitated by the increasing computing power, numerical simulation techniques, exemplified by density functional theory (DFT), have been applied to assist the investigation of the oxidation process, which involves the FAOR on different catalyst surfaces [54–59], temperature [60], pH value [61] and elastic strain [62]. For example, DFT calculations were employed to investigate the underlying mechanism of FAOR over different surfaces of PdCu with different Pd/Cu ratios. The results show Cu atoms of Pd_3_Cu donate the electrons to Pd atoms with the trimer to realize the bimetallic synergetic effect. Therefore, Pd_3_Cu reduces the adsorption ability of CO and enhances the ability of anti-CO position, thus increasing the activity and stability.

### D-Band Center and Volcano Plot

The reaction rate of FAOR is significantly contributed by the adsorption of intermediates on the catalyst surface, which is the basement for the catalyst design. Sabatier theory indicates the ideal catalyst should bind the reactant with a medium strength [63]. Too strong strength causes difficulty in desorption of products, while a too weak bond is unable to activate reactants. In the case of FAOR, the binding between the formate intermediate and platinum is considered through the Pt-O bond. Strong adsorption would lead to an increase in formate coverage, and a decline in oxidation rate, probably because it is difficult for the too closely packed bidentate formate to convert to the reactive specie monodentate [51]. Besides, a weaker metal–oxygen bond scission requires less energy. These all make improved catalytic activity when decreasing adsorption energy. However, as the bond strength further shifts away from the optimum value, the formation of formate becomes too weak and restricts the follow-up reactions, also causing a decrease in performance [64, 65].

**Fig. 5:**
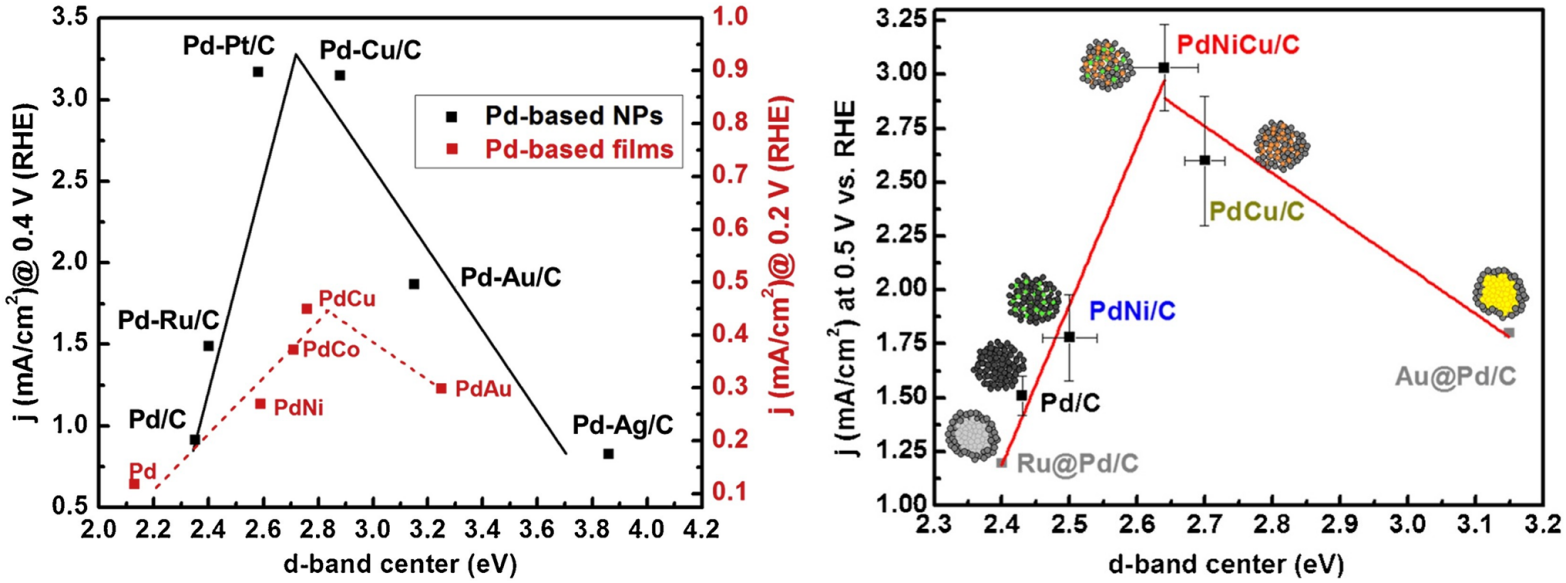
Volcano plots of catalytic activity toward the formic acid oxidation reaction (FAOR) vs d-band center for various catalysts [71, 73]

In order to accurately describe the relationship between the bond strength and catalytic activity, Hammer and Nørskov developed the “d-band center model,” in which the electronic structure of the catalyst could be calculated through the weighted mean energy of its d-band, namely d-band center, which correlated to the binding strength [66–68]. Catalytic reactivity, therefore, could be controlled through various methods to optimize the position of the d-band center which is called electronic effects [69]. In the application of catalyst design for FAOR, the d-band center is usually adjusted by alloying through the two main mechanisms: (i) The introduction of a second element can lead to the migration of charges if they have unequal electronegativity. This factor is named ligand effect [70]; (ii) besides, another influence is contributed by the change of lattice constant due to the lattice mismatch. This effect could cause strain on the catalyst surface, thus referred as strain effect [71, 72]. Both effects can cause redistribution of value electrons and shift the d-band center. A great quantitative implementation of the classical Sabatier theory and electronic effects is the volcano plots, which demonstrate the optimized d-band center and “best” catalyst. A series of experiments were conducted by Hu and co-workers to investigate the effect of different transition metals on the d-band center of Pd-based catalysts, and their catalytic activities toward FAOR [65, 71, 73]. The d-band center of each alloy was calculated through the integration of the normalized first moment of the density of state (DOS), and all showed a typical volcano plot with their performance in those studies (Fig. 5).

In general, after long-term research on FAOR and resulting from the rapid development of electrochemical measurement methodologies, particularly in situ characterization techniques, there has been now a profound understanding of the mechanisms underlying FAOR. Except for the widely accepted dual pathway theory, current research primarily focuses on the study of the reaction intermediate formate. In this context, computational simulation techniques, such as DFT, have provided substantial support. However, most simulations are based only on simplistic models. For instance, DFT calculations necessitate the specification of particular crystal facets. Yet, practical catalysts often possess complex structures. Even single crystals may have high-index facets on the surfaces, where active sites are usually located. Therefore, significant efforts are still required in order to build model for providing further understanding about the reaction mechanisms with multicomponent alloy and complex nanostructures.

## Nano Scale: Catalyst Design and Synthesis

The studies of the mechanisms provide a fundamental understanding for FAOR. Armed with these mechanistic insights, we now pivot our attention toward the consequential endeavor of the catalyst design. Drawing inspiration from the elucidated reaction mechanisms, researchers were able to propose different strategies for designing new catalysts, in order to obtain desirable catalytic pathways and mitigate undesired side reactions. Moreover, through intricate manipulations at the atomic and molecular levels, the catalyst composition, structure and morphology were manipulated to achieve designed strategies. This section will summarize common strategies for enhancing catalytic activity and stability reported in recent studies.

### Strategies of Catalyst Design for FAOR

The design of FAOR catalysts is mainly optimized from two aspects. One is to improve catalytic activity, ensuring that formic acid can be rapidly and completely oxidized on the catalyst surface. On the other hand, it is also necessary to consider their stability, that is, the catalytic activity degrades under long-term operation.

There are a number of studies that contribute to the improvement of FAOR activity, and the methods could be classified into two strategic factors. The first is increasing the catalytic active sites of a catalyst. Benefiting from the rapid development of nanomaterial synthesis technology, structural engineering has been significantly deployed in catalyst design. Many unique nanostructures, even the single atom, were reported, which showed a large specific surface area and high performance. In addition to pursuing more active sites, boosting the intrinsic activity of each site is another important strategy. As discussed in the section of the FAOR mechanism in the last section, the catalytic activity is strongly dependent on the binding strength between the catalyst surface and reactants/products. Both numerical and experimental results show that alloying and/or support could adjust the electronic structure of the catalyst, thus affecting the binding strength toward different reactions.

#### Structure Effect and Crystal Facets

**Fig. 6:**
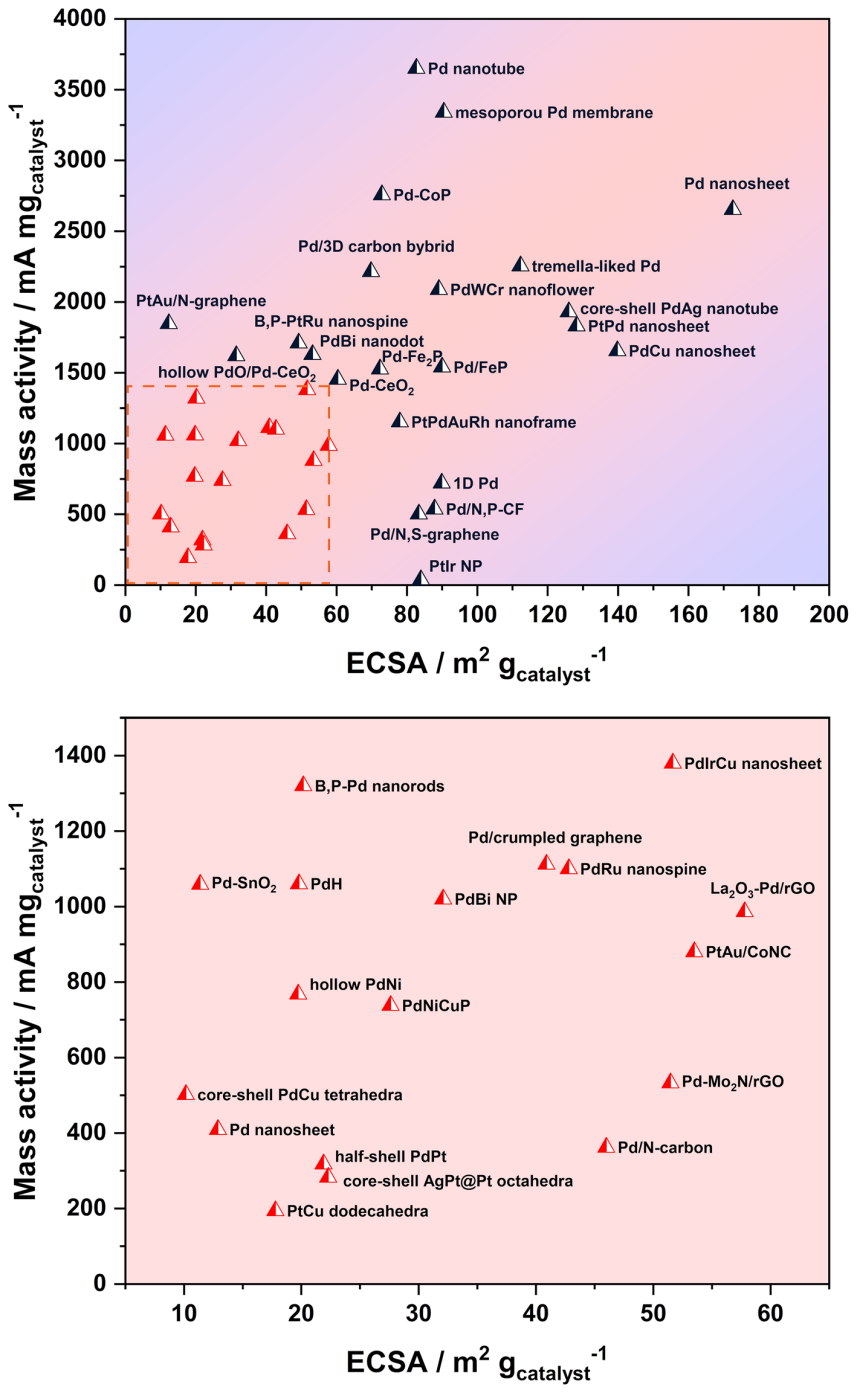
Mass activity vs electrochemical surface area (ECSA) for different catalysts toward the formic acid oxidation reaction (FAOR) reaction reported in recent studies. Partially enlarged view of the square area shown in bottom

Structure engineering is a powerful tool that could be applied in catalyst development. Figure 6 summarizes typical catalysts reported for FAOR in recent studies, with the mass activity shown vs the ECSA of the catalysts toward FAOR. A nearly linear relationship is obtained for the mass activity demonstrated as a function of ECSA. This is similar to the catalysts prepared for ORR, where a similar relationship between activity and ECSA was also reported [74–76]. This can be ascribed to the more active sites provided with a higher ECSA. In this case, the synthesis of the catalyst with a high surface area received significant attention in developing highly active catalysts. In the catalyst synthesis process, a high surface area can be achieved through creating rough surfaces and even pores with solid catalysts with different dimensions [77], such as nanoframes, nanotubes and nanosheets. In addition, with structure engineering, the formation of high-energy sites could be facilitated, such as crystal defects and crystal/amorphous interface [78], which can significantly promote the catalytic activity for FAOR even with the same ECSA. In the following parts, these will be discussed in detail.

#### *0-Dimensional* (0D) Catalysts with Special Structure

**Fig. 7:**
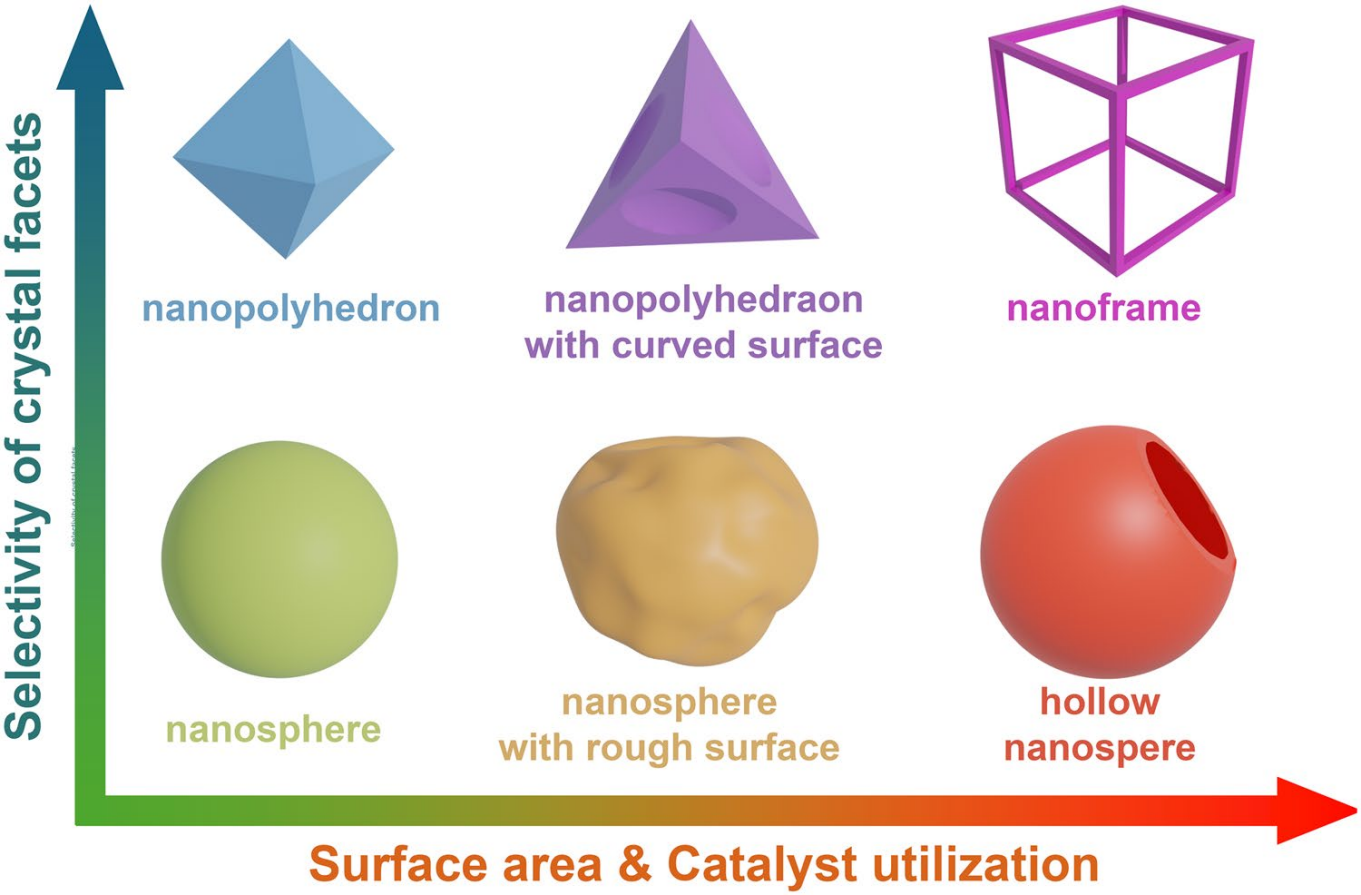
Key advances of the structural engineering research to improve the activity of 0D catalysts toward the FAOR

0D catalysts, represented by Pt and Pd nanoparticles supported on carbon nanospheres (Pt/C and Pd/C), are widely used in fuel cells. Despite their activities reported in recent studies were not as competitive as other nanostructures discussed later, they still received great attention toward FAOR due to the easy synthesis process. However, these conventional 0D nanoparticle catalysts suffer from various intrinsic drawbacks, including low activity and poor stability. Therefore, there were some attempts to modify the nanoparticle catalysts in recently reported studies, in which pursuing the larger surface area was their main target as it could offer more active sites for FAOR. These attempts are summarized in Fig. 7. They can be categorized into two approaches, including simply creating a larger specific surface area and controlling the exposed crystal facets.

To achieve a high specific surface area, the hollow nanostructure is one of the most popular strategies which could be achieved by introducing a solid spherical template, such as carbon nanospheres, and bubble templates (NH_3_ or CO_2_). The hollow nanostructure has a significantly increased surface to volume ratio compared with a conventional solid nanoparticle. Chen and co-workers synthesized PdNi bimetallic hollow nanocrystals with a dendritic shell [79]. The rough surface of the prepared catalysts is comprised of a great number of dendrite-like PdNi nanoparticles, thus providing a high surface area. These factors endow the prepared catalyst with an improved ECSA (19.74 m^2^ g_Pd_^−1^), which is 2.1 times higher than that of the commercial Pd/C catalyst. A similar study was reported for a porous PdPt half-shell catalyst [80]; the ECSA of 21.3 m^2^ g^−1^ was obtained. In addition to the benefit of the hollow structure and rough surface, the researchers created a mass of small pores and crystal defects (such as distortions, twin boundaries and atomic holes) on the shell. These rich surface defects serve as the catalytically active sites, thus further improving ECSA of the catalyst. Besides, the mass diffusion is also enhanced through the porous shell, because reagents, such as formic acid, can transport to the active sites through these pores. A PdO/Pd-CeO_2_ nanocatalyst with a similar hollow structure was also reported, showing an excellent catalytic activity of 1.62 A mg_Pd_^−1^ at 0.68 V toward FAOR [81]. Recently, a hollow high-entropy alloy, involving Pd, Cu, Mo, Ni and Co, was also reported for ORR and FAOR [82].

Boosting FAOR with designed crystal facets is another important methodology, which is well demonstrated for electrocatalysts with polyhedral nanostructures. Previous research has demonstrated the catalytic activity toward FAOR varies on different crystal facets [83]. This difference is mainly contributed by the onset potential for the oxidation of the adsorbed intermediates on different facets. Polyhedral structures, such as tetrahedrons [84], cubes [85] and decahedrons [86], that enclosed by the specific crystal facets have been demonstrated. The exposed surface required for the FAOR, therefore, could be achieved by controlling the structures of the synthesized catalysts.

In recent studies, the polyhedral nanostructures with high surface area attract more interest since they have the advantages of both strategies, i.e., large surface area and controllably exposed crystal facets. These catalysts were usually achieved by creating porous, curved surfaces or even framework structures through galvanic replacement or chemical etching of polyhedral structures. A core–shell CuPd@Pd catalyst with a concave tetrahedral structure that was introduced in Chen and co-workers’ study [77]. Benefiting from its concave surface, the ECSA of the prepared catalyst reached 10.17 m^2^ g_Pd_^−1^, compared with 5.98 m^2^ g_Pd_^−1^ of the benchmark JM Pd black. Jiang and co-workers reported an octahedral AgPt@Pt nanocatalyst with a porous surface structure [87]. This structure was achieved by selective etching of Ag segments using HNO_3_ from an AgPt octahedral nanostructure, exposing inner Pt atoms and leading to an enriched Pt surface. Consequently, the as-prepared AgPt@Pt catalyst demonstrated a mass activity of 10.8-fold larger than that of the commercial Pt black.

Furthermore, the nanoframework is considered as a structure that can further optimize the utilization efficiency of catalysts, therefore, receiving great interest in recent years. The PtPdCu nanoframe was prepared from PtPdCu nanocubes by selectively etching the Pd-rich cores with FeCl_3_ solution and HCl [88]. This designed framework structure demonstrated multiple merits toward FAOR. The maximized number of active sites and abundant stepped atoms produced during the etching process offers a large specific surface area, while the three-dimensional structure of the nanoframe provides a pathway facilitating the access of the reaction species to the catalyst surface. Frames with other polyhedral structures were also reported. They all demonstrated improved catalytic activities contributed by the large surface area and optimized charge and mass transfer. For example, Wang and co-workers presented a dodecahedral PtCu nanoframe catalyst for FAOR through a similar strategy [89]. The preparation of PtPdRhAg nanoframes with an octahedral structure and small particle size of less than 6.5 nm was introduced in Saleem and co-workers’ study [90]. A cubic PdAg catalyst with a framework structure was also reported [91].

#### *One-Dimensional* (1D) Nanostructures

Fuel cell catalysts with 1D nanostructures, such as nanowires, nanorods, nanotubes and nanochains [92–95], have also received increasing prominence in recent studies. Researchers from our group have also well-reviewed the 1D electrocatalysts and their application for the oxygen reduction reaction (ORR) as well as the hydrocarbon oxidation (including formic acid, methanol and ethanol) [96–98]. Compared with nanoparticles, 1D nanostructures show good potential to alleviate the inherent drawback resulting from aggregation, dissolution and Oswald ripening [99].

It has been well demonstrated that single-crystal 1D nanostructures can facilitate electrocatalytic activity via exposing highly active crystal facets, along with promoting electron transport through the path directing effect [97]. The study reported by Jiang and co-workers demonstrated the preparation of PtAg nanowires and its application for FAOR [100]. They noted the formed nanowires had an oriented attachment along the < 111 > direction due to the adsorption of polymer structure-directing agent on (110) and (100) facets during the catalyst synthesis process. Previous work based on both experimental and numerical calculation methods for investigating the FAOR mechanism has concluded that the CO formation reaction on the Pt(111) surface was more difficult than others [50]. Therefore, the dominant Pt(111) facets within the PtAg nanowires are favorable for suppressing the indirect pathway of FAOR and boosting the overall catalytic efficiency. A similar crystal facet effect was also observed in a study of trimetallic AuPtRh nanowires [101]. Besides, the nanowire structure was achieved with various Pt-based alloys, such as PtRu [102], PtAu [103] and PtPd [104]. Moreover, the benefit of exposing high-active facets was also well demonstrated with the Pd-based nanowires, where higher performance was contributed from the rapid FAOR on Pd(100) facet [83, 105].

**Fig. 8:**
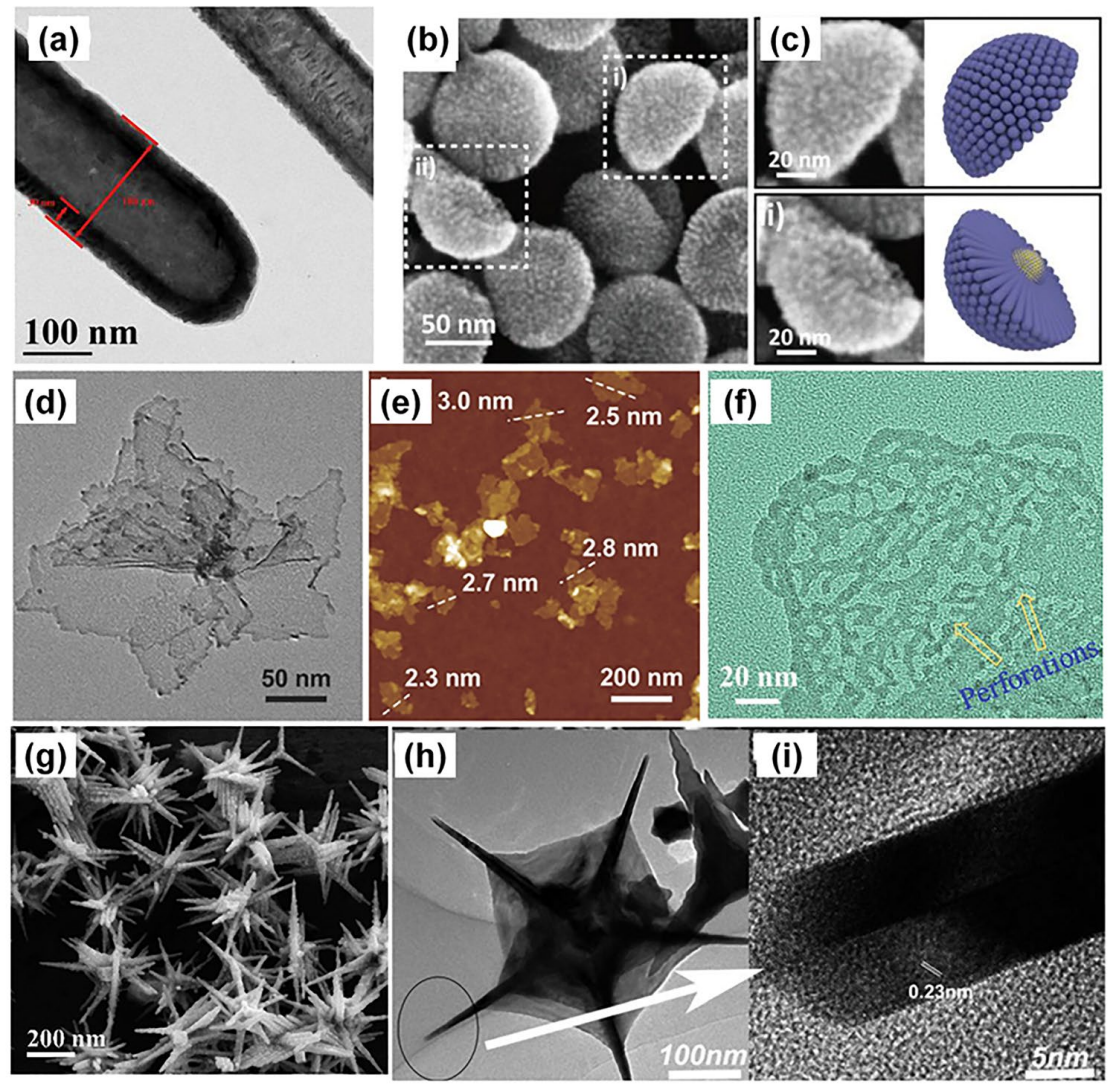
Typical nanostructures of catalysts for the formic acid oxidation (FAOR). **a** A TEM image of PdAg@Pd core–shell nanotubes [106]. **b, c** SEM images of Au@PtPd hemispherical nanostructures [107]. **d, e** TEM and AFM images of ultrathin PdCu alloy nanosheets. [108] **f** A TEM image of perforated Pd nanosheets [109]. **g** A SEM image of hyperbranched PdRu nanospine [110]. **h, i** TEM images of web-like **g** Pt nanopentagons [111]

Inspired by the excellent catalytic activities of the hollow and porous 0D nanocatalysts, hollow nanowires, or called nanotubes, have also been frequently reported as they can further increase the surface area and provide more active sites as well. For example, an enhanced ECSA of 63 m^2^ g^−1^ was measured in a study of PdAg@Pd core–shell nanotubes (Fig. 8a) [106]. Moreover, the mesoporous Pd nanotubes were demonstrated in Ding and co-workers’ experiments [95]. Besides the high specific surface area provided by the tube structure, the presence of the soft template randomly perturbs the nanotube growth and generates mesopores and anisotropic substructures (such as lattice defects and step edges), which offer more additional active sites and high-energy domains to accelerate the adsorption and oxidation of formic acid.

The 1D nanostructure could be further assembled to form high-dimensional materials, such as chains [112] and networks [113, 114]. Very recently, a hemispherical nanocatalyst assembled by PtPd nanowires (Fig. 8c, d) was reported by Liang and co-workers [107]. In this study, the anisotropic 1D catalysts were formed on Au seeds, enabling the nanostructure to have a large specific surface area and thus demonstrating excellent electrocatalytic activity for the oxidation of various liquid fuels (methanol, ethanol and formic acid).

#### *Two-Dimensional* (2D) Nanostructures

2D nanomaterials have received increasing interest since the report of graphene in 2004 [115]. In the studies on FAOR, the nanosheet, as a typical 2D nanomaterial, demonstrated the largest surface area and best catalyst activity through a comparison of the mass activity of different nanostructures (Fig. 6), thus has been widely reported recently for FAOR application. The ultrathin 2D structure can expose more interior atoms, thus providing a relatively high surface to volume ratio. This structure is usually formed with the assistance of carbon monoxide (CO), as the CO can strongly bind to the metal (111) facets and induce 2D growth. In Yang and co-workers’ work, the ultrathin PdCu alloy nanosheets with a thickness of 2.8 nm were prepared as the highly efficient electrocatalyst for FAOR (Fig. 8d, e), in which an enlarged ECSA of 139.8 m^2^ g_Pd_^−1^ was achieved [108]. A similar nanostructure was also obtained in the research of PtPd alloy from another group, showing an almost tenfold higher ECSA compared with a JM 20 wt% Pd/C catalyst [116], reaching up to 128.23 m^2^ g^−1^, delivering a mass activity of 1.831 A mg^−1^.

Besides, the advantage of the porous structure was also applied to these 2D nanomaterials. Zhang and co-workers synthesized perforated Pd nanosheets with a crystalline/amorphous heterostructure [109]. As shown in Fig. 8f, they noted that the perforated structure not only reduced the catalyst usage amount and improved utilization, but also provided more active sites due to increased atomic edges and steps, as mentioned above. As a consequence, the ECSA measured in this study reached 172.6 m^2^ g_Pd_^−1^, which is the highest value compared with other reported Pd-based catalysts in the literature. In addition, a Pd nanosheet with a large porosity was also synthesized through a one-pot hydrothermal method in Qiu and co-workers’ report, in which the nanosheet structure was knitted by interweaved ultrathin nanowires [117]. The researcher demonstrated that besides the properties of the nanosheet, the nanowire structure further facilitated the mass transport and charge transfer, which was revealed by the high exchange current density (114.8 A g^−1^) with the obtained Tafel plots.

The preparation of a 2D Pd electrocatalyst with periodically ordered mesoporosity was also reported recently through the assistance of a lyotropic liquid–crystal template [118]. This periodically ordered structure was considered to be able to optimize some congenital shortcomings of conventional porous nanosheets, for example, the sluggish mass transfer can be caused by the pores that are tortuous and isolated from each other, which can further lead to a lack of fresh reactants deep inside and, therefore, rapid catalyst poisoning. In this study, the periodically ordered catalyst was precisely synthesized by controlling the potential and temperature of electrodeposition, demonstrating great electrocatalytic activity (3.34 A mg^−1^) for the FAOR compared with a commercial 30 wt% Pd/C catalyst (0.43 A mg^−1^).

Furthermore, these 2D nanosheets can also be further assembled into a 3D structure. A typical example was shown in Zhang and co-workers’ study, where a layered and heterostructured Pd/PdWCr nanoflower was reported with a high mass activity of 2.09 A mg_Pd−1_ recorded in the measurement in 0.5 M H_2_SO_4_ + 0.5 M HCOOH aqueous electrolyte [119].

#### Nanostructures with *High-index* Facets

Most of the conventional structures discussed above only involve low-index crystal facets, such as {111} and {100} facets. Some works also reported the attempts to expose high-index facets with nanostructured catalysts as they usually provide higher catalytic activity for FAOR, which are contributed by the presence of the high density of atomic steps, edges, kinks and dangling bonds on the surface [120]. The DFT calculation reveals FAOR on the high-index facets is dominated by the direct pathway, which can suppress the formation of poisoned intermediates and provide high-performance electrocatalysts for the DFAFC [121]. Because high-index facets are most shown in high-curvature structures of Pd and Pt catalysts, such as the spine tip, these sharp structures are also considered to concentrate electric fields at the surface and facilitate catalytic activity through field-induced reagent concentration [122]. Wang and co-workers reported the preparation of hyperbranched PdRu nanospines (Fig. 8g) with a 2.8-fold ECSA compared with that of Pd nanoparticles [110]. In their further study, the mass activity of the PdRu nanospines reached 1.37 A mg_Pd_^−1^ after doping boron (B) and phosphorus (P) [123]. In addition, a web-like Pt nanopentagon (Fig. 8h, i) with sharp branches was demonstrated in Lai and co-workers’ research, in which the authors noted that the high-index facet (554) plane presented at the boundary of the branch surface [111]. The mass peak current density of 739 mA mg^−1^ was recorded, which is 1.7, 6.8 and 23 times higher than that of commercial Pd/C, Pt/C and Pt black. A similar urchin-liked nanostructure was also reported for PdCu [124] and PdCuPt catalysts [125].

#### Single Atom Catalysts (SAC)

In the previous section, we discussed that electrocatalytic performance demonstrated a linear relationship with their ECSA. Consequently, extensive research has focused on synthesizing highly efficient catalysts. Simple mathematical calculations reveal that reducing the catalyst size can increase surface area, thus enhancing utilization. Significant effort has been dedicated to studying ultrasmall catalyst particles [126]. The most extreme case is the single-atom catalysts (SACs), which, with nearly 100% utilization, has attracted considerable attention. For example, Liu and co-workers synthesized Pt SAC on Au nanocrystals, achieving an impressive FAOR catalytic activity of 38.6 A cm^−2^, 370 times greater than that of conventional Pt/C catalysts [127]. Similar electrocatalysts have also been reported on titanium nitride supports [128] and hollow carbon nanorods [129].

Furthermore, SACs typically exhibit distinct electrochemical properties and reaction pathways compared to conventional nanoparticle catalysts. This is because the metal sites in SACs usually carry a partial positive charge, resulting in reduced electron density and thus altering the metal–reactant interactions. More importantly, the spatial isolation of metal atoms in SACs can be exploited to modify the adsorption configuration of reactive intermediates and prevent side reactions that require adjacent metal sites. Based on this principle, Xiong and co-workers reported a single-atom Rh/N-doped carbon electrocatalyst for FAOR [130]. DFT calculations indicate that this catalyst possesses a high barrier for CO formation and an unfavorable binding with CO, thus exhibiting excellent CO tolerance. A similar strategy has also been reported with an iridium single-atom catalyst on nitrogen-doped carbon [131].

#### Alloying

Alloying is another crucial strategy that has been widely applied in catalyst design. The catalytic activity toward FAOR, as discussed in the mechanism section, is highly dependent on the binding strength between the reactants/intermediate species and catalyst. Alloying provides an effective pathway to adjust this strength through the modification of the electronic structure of catalysts (electronic effects), which is contributed by the migration of charges (ligand effect) and lattice mismatch (strain effect). Previous work explored the correlation of FAOR catalytic activity and electronic properties of various Pd-based nanoparticles, including Ru, Pt, Cu, Au and Ag [71]. XPS analysis results revealed the shift of core level binding energy for Pd followed the order: PdRu < PdPt < PdCu < PdAu < PdAg, which well fit the simulation results in DFT. A typical volcano plot (shown in Fig. 5a) was demonstrated in this study where the maximum catalytic activity was located between 2.58 eV (PdPt) and 2.85 eV (PdCu) of the d-band center. This trend is similar to their previous results reported for the Pd-based bimetallic thin films [65].

**Fig. 9:**
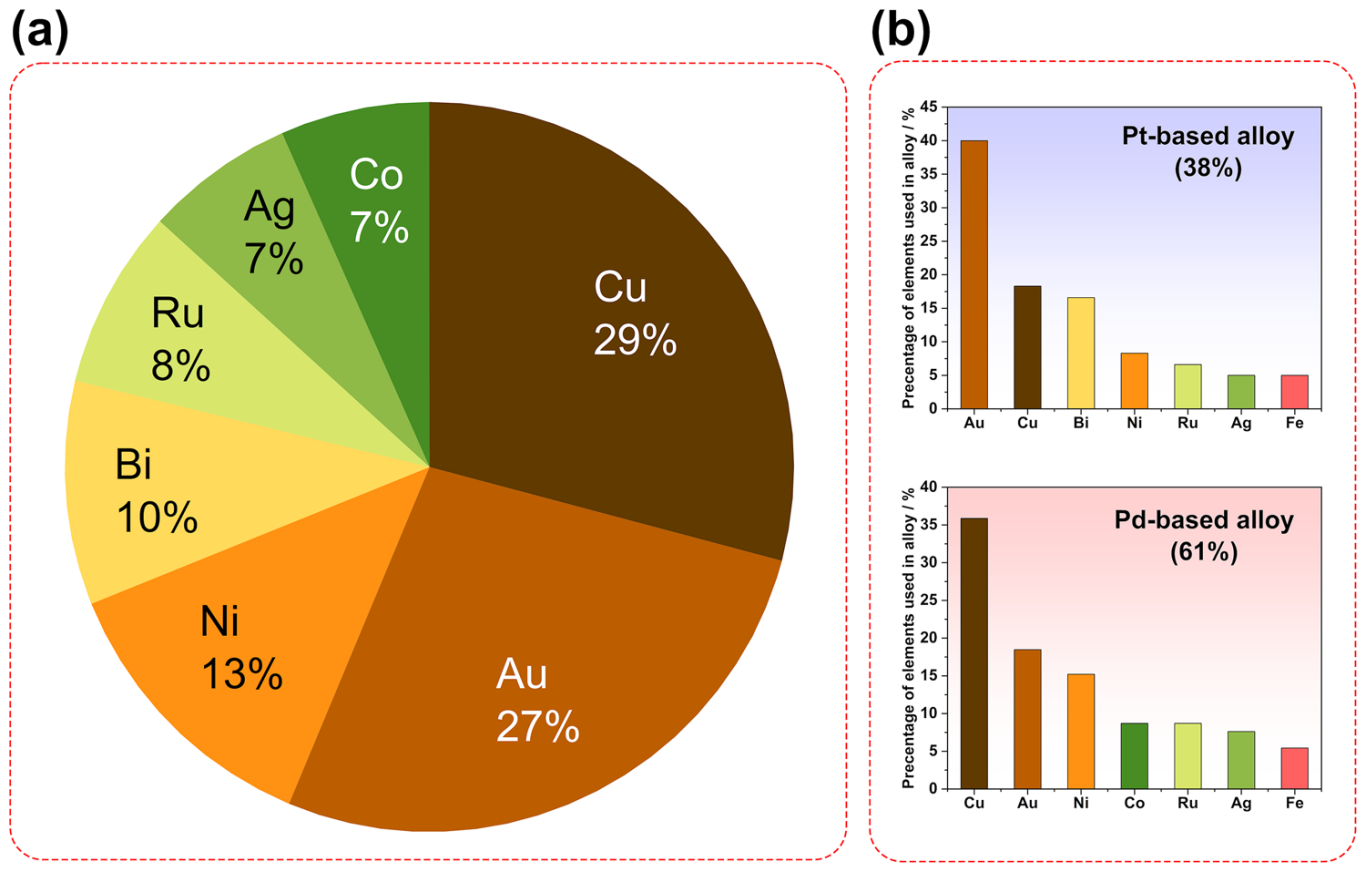
Percentage of different elements used in alloyed catalysts toward the formic acid oxidation reaction (FAOR) in recent studies. **a** Alloying elements. **b** Pt- and Pd-based catalysts

Although many computational and experimental studies have been conducted to identify the “best alloy” for FAOR, an obvious relationship between the catalytic activity and the electron structure of the alloy is still unclear. A number of metals, therefore, have been explored to be alloyed with Pt or Pd. Figure 9a summarizes the most common elements used to alloy electrocatalysts reported in recent studies. It is noted that Au and Cu share almost the same proportion, and they account for more than 50% of the total studies. However, when separately analyzing the usage of these elements in the Pt and Pd catalysts (Fig. 9b), it is obvious more investigations have been performed on Cu for Pd-based catalysts, while more to alloy Pt catalysts with Au. With the Pt catalyst, the biggest challenge is its indirect oxidation pathway toward FAOR. PtAu demonstrates excellent performance in suppressing the dehydration reaction and formation of poisoning intermediates, thus receiving the most interest in recent studies. Cyclic voltammetry (CV) measurements conducted on the PtAu/C catalyst revealed alloying with Au suppressed the indirect oxidation reaction by comparing the ratio of the first to second oxidation peak of the CV plot [132]. DFT calculation reported by Fan and co-workers proposed another reason to explain the dominated direct pathway with the PtAu catalyst from the contribution of energy barriers [133]. They noted the barriers of C-H bond cleavage were lower than that of the O–H bond on the Pt surface. The initial C-H bond activation governed the oxidation process through an indirect way. The introduction of neighboring Au could significantly reduce the barrier of O–H cleavage, thus facilitating the direct oxidation. A similar mechanism was also reported in the study of FAOR on the PdPb catalyst [134].

On the other hand, the excellent intrinsic properties of the Pd catalyst lead to its dominant direct oxidation. Compared to Pt, the high binding energy between Pd and formate causes higher formate anions coverage, thus blocking the ensemble site necessary for CO formation [135]. This enables its research efforts domain (61% of total studies, as shown in Fig. 9c), compared with Pt-based alloy (38%). Therefore, most of the studies about Pd focused on the synthesis of catalysts with controlled shapes, especially for the high surface area, as mentioned above. To achieve this aim, dealloying has been considered as the most effective method, in which a second metal as the sacrifice phase is reduced along with Pd and then removed to create rough surface. Copper, due to its low cost, easily to be reduced and removed, was widely used in the fabrication of Pd-based catalysts [89, 136]. The introduction of Ag can also serve as the sacrificial phase to obtain high surface area due to its high standard reduction potential (galvanic replacement) and easy to be leached away (i.e., by acid treatment) [87, 91, 100, 106, 137].

In addition to the electronic structure modification, the introduction of other elements can also provide extra functional groups, such as hydroxyl ion (-OH_ad_), to promote the oxidation of the poisoning intermediates. A typical example was demonstrated for a AuPtRh catalyst [101]. In this study, with the introduction of Rh atoms, although no shift was observed in Pt 4*f* peak through the high-resolution XPS analysis (Pt and Rh have the same electronegativity, X_Pt_ = X_Rh_ = 2.28), the negative shift of the onset peak in the CO stripping measurement suggested easier CO removal on the catalyst surface. The researchers ascribed this to the -OH_ad_ generated from the extra Rh atoms, instead of electronic effects. Besides, the negatively shifted peak in the CV plot of the FAOR also reflected the contribution from hydroxyl species as it enabled the oxidation at a much lower potential [77].

The formation of poisoning CO on the Pt surface requires at least three continuous Pt atoms. The presence of a second metal, therefore, can disrupt this continuous distribution and suppress the indirect oxidation of formic acid. This is defined as the “third body effect” [138]. Choi and co-workers demonstrated a facile method to modify the Pt/C catalyst by irreversibly adsorbing Bi atoms on the catalyst surface [23]. The authors noted that the adsorbed Bi served as the third body and promoted FAOR through the direct pathway, thus leading to an improved peak power density to around 180 mW cm^−2^ in the single-cell test, which is 2.85 times higher than the value of the non-modified Pt/C catalyst. This strategy was also applied to the Pd catalyst and was further extended. The study reported by Shen and co-workers indicated, compared with the random alloy with a disordered structure, ordered intermetallic could further isolate catalyst atoms and limit the formation of the CO intermediate [139]. Based on this mechanism, ordered PdBi alloy was synthesized and recorded a mass activity of 1.02 A mg_Pd_^−1^ in the electrochemical measurement in N_2_-saturated 0.5 M H_2_SO_4_ + 0.5 M HCOOH electrolyte while the disorder PtBi catalyst only reached 0.4 A mg_Pd_^−1^.

Last, one thing that is worth stressing is the FAOR mechanism is still under debation and development. More numerical calculations are being conducted to guide further practical experiments. For example, recently, research reported by Sui and co-workers predicted the catalytic activity of several Pd-based alloys based on their carbophilicity and oxophilicity through the DFT calculation [140]. Among them, Mo@Pd(111) demonstrated the strongest *O–H binding and weakest *CO binding, therefore being considered the most promising bimetallic Pd-based catalyst for FAOR.

In addition to the conventional alloying method, there are some other attempts that have been reported recently to introduce a second phase for boosting FAOR. For example, metal oxides have been frequently reported in recent studies, including TiO_2_ [141], MnO [142, 143], Fe_3_O_4_ [144], La_2_O_3_ [145] and CoO [146]. Among them, the decoration of SnO_2_ on Pd nanocubes was demonstrated in Rettenmaier and co-workers’ study [147]. A negatively shifted peak was observed in the CO stripping measurement, suggesting a weaker Pd-CO bond formed when SnO_2_ was present. Xu and co-workers reported a doping technique using boron (B) and phosphorus (P) to modify RdRu nanospines [110, 123] and Pd nanorods [148]. The B, P doping not only downshifted the d-band center position, but also facilitated the formation of the oxidized boron and oxidized phosphorus species, which promoted the oxidation of adsorbed oxygenated species. The metal(-transition) phosphide system also received attention, because it can effectively regulate the electronic structure and induct catalysts to form different valences due to strong P-metal interaction [149]. For instance, Pd/FeP catalyst was published in Bao and co-workers’ research [150]. In this work, the XRD and XPS analyses revealed the catalyst existing in the form of metallic Pd and PdO. This combination of metal and oxides on the catalyst surface has also been reported, showing improved catalytic activity in previous studies [151]. A similar strategy was used to prepare Pd/CoP catalysts for FAOR, for which a great peak power density of 150 mW cm^−2^ was recorded in the DFAFC single-cell test [152]. Very recently, a few attempts were reported, in which the palladium hydrides (PdH) were used for catalyzing FAOR, and also showed the ability to tune the electronic structure and suppress the CO* generation or bonding [153, 154].

#### Support Effect

The contribution from the support to catalysts comes from several aspects. The most intuitive benefit is optimizing morphology, including catalyst dispersity and particle size, because of the interaction between the metal phase and support. The most commonly reported catalyst supports are carbonaceous and CeO_2_.

Benefiting from the great electrical conductivity and large surface area, carbonaceous materials are widely recognized as excellent catalyst supports in fuel cell applications, including carbon black [155], carbon nanofibers [156], carbon nanotubes and graphene [157–162]. Among them, graphene has received the most interest in recent decades due to its extremely high specific surface area and excellent electrical conductivity. Xu and co-workers reported N-graphene supported PtAu, for which small charge resistance revealed by the electrochemical impedance spectroscopy (EIS) analysis, suggested this support provided a multidirectional electron transfer route. The formed catalyst, therefore, exhibits an excellent mass activity of 1.847 A mg^−1^ toward FAOR [158]. Zhou and co-workers demonstrated a three-dimensional crumpled graphene by the spray-drying method [159]. This 3D structure with the high specific area led to a small particle size and a more uniform distribution of the supported Pd catalyst. A 3D support was also shown in the study of graphene–carbon nitride hybrid [160]. In this study, an ultrasmall particle size of 3.6 nm was obtained, contributed by the anchoring effect from the support. Very recently, the low-cost chromium nitride (CrN) was reported to be utilized as a support for the loading of epitaxial ultrathin Pt atomic layers [163]. Benefiting from the strong anchoring and electronic regulation of Pt atomic layers by CrN, the synthesized electrocatalyst demonstrated excellent activity with a mass activity of 5.17 A mg_Pt_^−1^.

Besides, the strategy of doping was also deployed to the carbonaceous supports, including using boron (B) and nitrogen (N), showing enhanced properties for inducing the anchoring sites for improving catalyst distribution and electron transfer [158, 160]. Similar to the effect of the alloying, electronic metal–support interaction (EMSI) was used to describe the influence of catalytic activity from charge redistribution between the catalyst and support. For example, a Pd catalyst supported on Mo_2_N was demonstrated with a shifted binding energy in the XPS analysis, for which the electron density was enhanced through electron transfer from Mo_2_N to Pd [164]. A similar phenomenon was also observed on PdCu supported on WO_2.72_, but the electron transfer in this study was in the opposite direction from metal to support [165]. However, the negatively shifted peaks in the CO stripping measurement revealed that the ability to remove poisoning CO was boosted for both catalyst support combinations above.

Ceria (CeO_2_) has also been studied as the catalyst support material due to its contribution to optimizing electron transfer and surface oxygen mobility [166]. The DFT calculation revealed its properties of fast oxygen mobility could cause overdosed oxygens on the Pd surface and assist removal of strongly adsorbed poison species [167]. Besides, the migration of oxygen also promoted the formation of oxygen vacancies in the structure, producing high-active Ce^3+^/Ce^4+^ redox pairs. As a result, the catalytic performance was significantly enhanced by the electronic effect of the oxygen vacancies [168]. Zhang and co-workers demonstrated the catalytic activity of Pd/Pd-CeO_2_ hollow spheres toward FAOR [81]. In this study, the XPS analysis showed the presence of the two mentioned characteristic oxidation states of Ce, and the successful formation of the fresh catalyst surface during the in situ electrochemical reduction process. An excellent mass activity (1.62 A mg_Pd_^−1^), therefore, was recorded in N_2_-purged aqueous 0.5 M HCOOH + 0.5 M H_2_SO_4_ electrolyte.

#### Improvement of Catalyst Stability

The main mechanism of catalyst aging during FAOR is caused by poisoning and physical loss. The challenge of catalyst poisoning is caused by the adsorbed species on the catalyst surface, and gradually blocking its active sites [169]. Based on this fact, all the strategies discussed above, used to promote the direct oxidation and suppress the formation of poisoned intermediates (or facilitate their oxidation), can potentially promote catalyst stability.

In terms of physical loss, nanoparticles, the most common catalysts used for FAOR, suffer from dissolution, aggregation and Oswald ripening [99]. To overcome these challenges, various novel nanostructures have been proposed and synthesized to enhance catalytic stability. For example, different 2D nanosheets were reported in several studies [109, 116, 117, 170]. The TEM analysis showed these 2D morphologies could be essentially maintained after the long-term cycling tests. Based on the same mechanism, some catalysts with 3D nanostructures were also prepared with good durability for FAOR, such as nanoflowers [119], nanoshells [80] and networks [112]. Besides, some other nanostructures also show the ability to resist deformation, including nanotubes [95], nanoplates [111, 171] and nanospines [123].

Moreover, there are some other strategies that have been reported to increase the lifetime of FAOR catalysts. Yang and co-workers reported a stable Pd catalyst supported on a B-doped 3D carbon hybrid [160]. In this study, the catalyst stabilization mechanism was investigated by comparing the commercial Pd/C and the prepared catalyst using IL-TEM characterization. After 250 CV cycles of testing, many Pd particles were found detached from the carbon support for the Pd/C catalyst, and formed larger particles due to agglomeration and migration. However, only a slight change was observed in the particle size of the Pd/B-doped 3D carbon hybrid. The researcher ascribed this improvement to the abundant doping species and functional groups on the catalyst surface, which served as anchors to form strong metal–support interaction and enhanced durability. Besides, metal-complex also shows the potential to stabilize FAOR catalysts. For example, the introduction of (2-[1-(Benzyloxyimino) ethyl] benzothiazole-к^2^N,N] dichloropalladium(II)) on the Pd catalyst was reported by EI-Nagar and co-workers [172]. This complex not only restricted the catalyst particle size growth during the synthesis leading to large ECSA, but also act as a shell outside the Pd nanoparticles, preventing aggregation and dissolution of the active sites. Similar metal-complex assemblies were also reported in other studies [173, 174].

### Preparation Methods of FAOR Electrocatalysts

The synthesis process determines the properties of the formed catalyst, thus playing a crucial role in the achievement of the designed strategies mentioned above. The reported methods can be generally classified into two methods: chemical reaction methods and electrochemical methods.

#### Chemical Reaction Methods

**Fig. 10:**
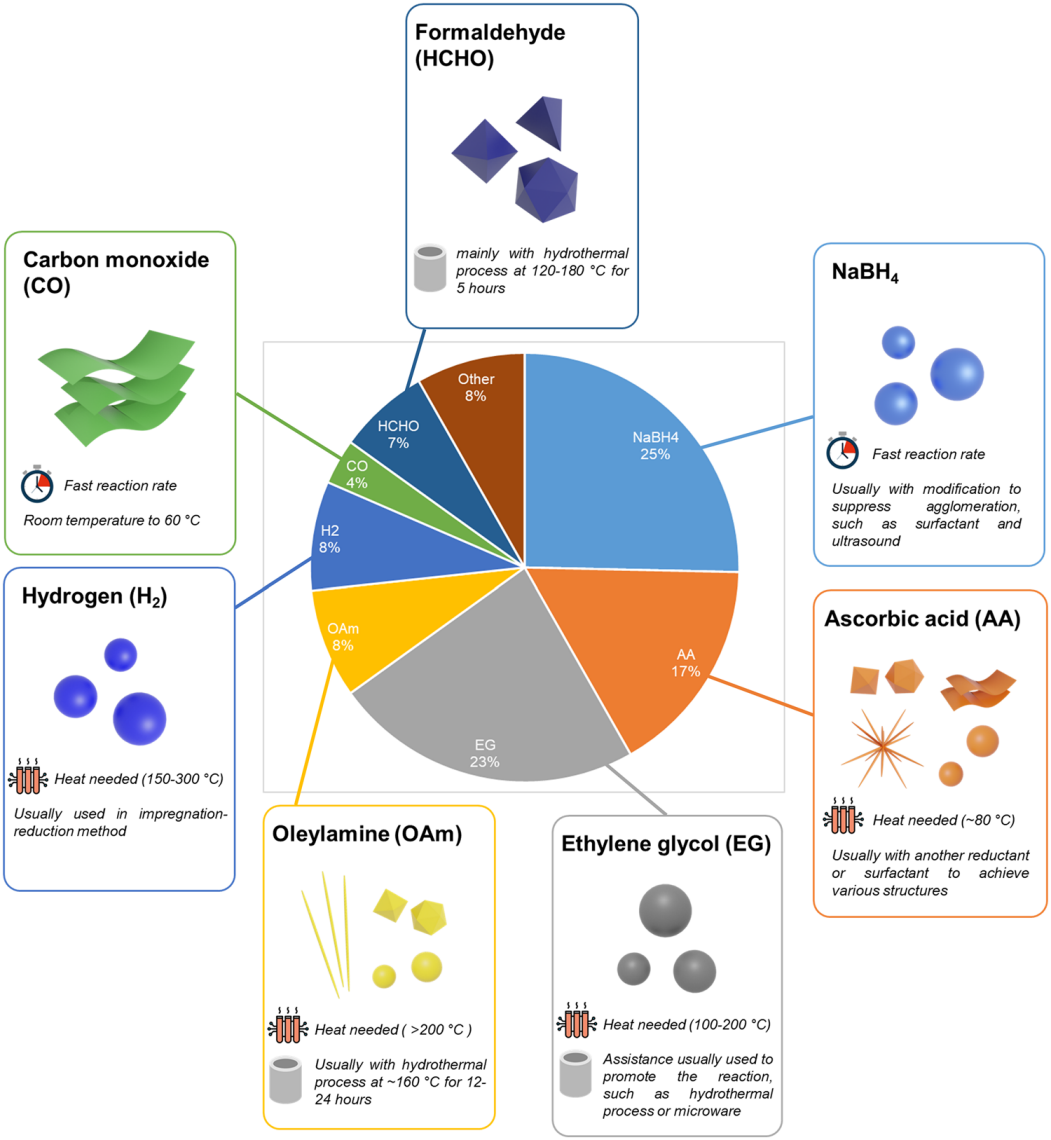
Proportion of the main reductants used for the synthesis of formic acid oxidation reaction (FAOR) catalysts and their main features

The chemical reaction method is the most common one for the preparation of FAOR catalysts. In a conventional process, catalyst precursors and reducing agents are mixed in an aqueous or organic solvent, and then catalysts are formed in solution or on support materials. Besides, extra assistance has also been applied in some studies, such as surfactant, microwave, heating and hydrothermal synthesis, to control the catalyst structure or reaction process. The choice of the reductant is mainly dependent on two aspects. (i) The elements of the catalyst. It is crucial to consider whether the reductant used in the synthesis has the ability to reduce all precursors completely. (ii) The expected nanostructure of the catalyst. In order to obtain the designed nanostructure, the reductant and necessary assistance need to be considered [175]. Based on these points, the common reductants used in recent studies to prepare FAOR catalysts will be discussed, and their deployment proportion in the synthesis and their features are summarized in Fig. 10.

#### Sodium Borohydride

Sodium borohydride (NaBH_4_) is a low-cost, strong reducing agent. A high temperature or long reaction time is usually not required for the process of the NaBH_4_ reduction method. Besides, as a strong reductant, NaBH_4_ demonstrates the most extensive practice and ability to reduce the most common metal precursors to synthesize catalysts. This leads to a quarter of the published works applying this method to prepare FAOR catalysts in recent studies.

NaBH_4_ is mainly employed in the reaction in the aqueous solution, and a buffer is usually required to create an alkaline environment. For example, in order to synthesize CNT-supported Pd for FAOR, Pd(NO_3_)_2_ as the precursor was first dissolved in water, and mixed with CNT by sonication, followed by adding NaBH_4_ into the mixture [176]. Using Na_2_CO_3_ solution as the buffer, Pd nanoparticles supported on three-dimensional crumpled graphene were prepared by using NaBH_4_ as the reducing agent [159]. A similar strategy was also applied to synthesize Mo_2_N/rGO supported Pd catalysts for FAOR [164]. Benefiting from its strong reducibility, NaBH_4_ shows an excellent ability in the reducing reactions, and thus has been widely used in the preparation of bi- or multimetallic catalysts [177–182]. In addition to the conventional carbon support, the preparation of catalysts on various other supports is also dominated by the NaBH_4_ reduction method, such as reduced graphene oxide (rGO) [183], carbon nitride [184, 185] and carbon bowls [186]. Furthermore, NaBH_4_ has also been used to modify commercial catalysts. Using NaBH_4_ to reduce Bi_2_O_3_, Bi was successfully introduced onto the surface of the commercial Pt/C catalyst [23]. The Bi-modified Pt/C shows an enhanced activity toward FAOR due to the “third body effect.”

The fast reaction rate allows researchers to prepare catalysts easily, but also leads to some drawbacks, such as poor distribution and heavy agglomeration. Some studies have attempted to address these issues. Among different strategies, surfactant assistance has received the most attention due to its low cost and easy integration with the NaBH_4_ reduction process. For example, a trimetallic catalyst PtAuRh was synthesized by a polyethylene glycol (PEG) assisted NaBH_4_ reduction method [101]. Precise reaction control has yielded catalyst nanowire structures. In addition to the use of surfactants, there are some other attempts reported. Fan and co-workers reported the preparation of PtAu nanoparticles by an ultrasound-assisted synthesis method [133]. Ultrasonic treatment was adopted to help achieve a uniform and dense catalyst loading on support without using any surfactant. The “water-in-oil” microemulsion method was also attempted in the fabrication of both Pt and Pd catalysts for FAOR. The water exists as the aqueous droplet in the oil phase, which can be used as individual nanoreactors where the catalyst precursors are only reduced by NaBH_4_ in these droplets. For example, by using cyclohexane solution as the oil phase, PdAu alloy nanoparticles were prepared. In this study, the researchers presented the controlled metal particle size with narrow size distribution [187]. A similar strategy was also used to fabricate PtAu nanocrystal. The prepared particles showed an average size of 4.8 nm, with higher monodispersity and smaller size compared with the catalyst formed in the aqueous solution [132]. Chemicals with a similar property to NaBH_4_, such as KBH_4_, have also been used to prepare FAOR catalysts [188, 189].

#### Polyol

Polyol has been widely reported in the preparation of electrocatalysts because it can serve as both solvent and reducing agent, which also makes the polyol reduction method low cost and low toxicity.

Ethylene glycol (EG) is the most common polyol used as the reducing agent, and its practice is second only to the NaBH_4_ in all reported chemical reduction methods. In its application, metal precursors are usually dissolved into EG and reduced at a temperature between 100 and 200 °C [190], for example, the preparation of PdCu on carbon support [191]. Metal precursors PdCl_2_ and CuCl_2_ were dissolved in EG to form a mixed solution, followed by adding carbon support the mixture. The mixture was further mixed with NaOH solution to adjust pH, then refluxed at 160 °C, which reduced precursors to micelles and produced the PdCu nanoparticles. The EG reduction method has been reported to form various catalysts, including the Pd [156, 192], Pt [86], PdBi [193] and PtPd [194], and on different carbonaceous supports, such as three-dimensional boron- and nitrogen-co-doped graphene aerogels (BNG) [195], nitrogen-doped carbon [155], nitrogen and sulfur dual-doped graphene [161] and 3D carbon sheets [196]. In addition to Pt- and Pd-based catalysts, there are some attempts at the preparation of other metals for FAOR by using EG as the reagent, such as Rh supported on graphene [157].

EG can serve as the solvent, reducing agent and surfactant, which makes it a popular method to prepare metal catalysts; however, the high reaction temperature limits its application. Some other assistant techniques, therefore, were proposed to avoid the direct heating of chemicals. A classic example is the microwave-assisted method. This has been used to prepare PdPb nanoparticles [197] and Pd/FeP [150]. A CoP/C supported Pd catalyst was also synthesized by a similar method, in which the particle size was well controlled below 5 nm [152]. Besides, the preparation of Pd catalysts supported on CeO_2_ [198], Fe_2_P/C [199], ZrO_2_/ MWCNT [200], WO_3_/C [201] and Pt on N, B-3D graphene aerogel [202] has also been reported. In addition, the hydrothermal technique is another method that could be used to accelerate the EG reduction method process [116, 203, 204]. Polyols with similar properties, including triethylene glycol (TEG) [205] and polyethylene glycol (PEG) [112], have also been reported for preparing FAOR electrocatalysts.

Oleylamine (OAm) is another chemical with great properties as it can also act as a solvent, reductant and surfactant for synthesizing FAOR catalysts. Xi and co-workers demonstrated the preparation of PdCu nanoparticles on WO_2.72_ nanorods for FAOR, by the OAm reduction method [165, 206]. The application of OAm could also be conducted with co-surfactants to prepare catalysts with various structures. Using OAm as the main reducing agent and borane tributylamine complex (BTB) or oleic acid (OAc) as the co-surfactant, Pd, PdCu and PdCu/WO_2.72_ were successively prepared [207]. The PdCu nanoparticles synthesized in the presence of OAm showed ultrasmall size, only around 3 nm. Web-like platinum nanopentagons were obtained through the hydrothermal synthesis method using OAm and tri-n-propylamine [111]. With hexamethylenetetramine (HMTA) and cetyltrimethylammonium bromide (CTAB), a dodecahedral PtCu nanocatalyst was formed using a similar strategy [89]. Other nanostructures, including nanowires [208, 209] and nanocubes [210], were also demonstrated.

The synthesis of FAOR catalysts has also been reported with other polyols. Ye and co-workers demonstrated the preparation of PtPdCu alloy nanoframes using DMF [88]. The metal precursors were first reduced in DMF with the assistance of capping agent polyvinylpyrrolidone (PVP) and KI to form concave nanocubes, followed by selectively etching to form nanoframes. Pd nanocubes and nanoparticles with high-index facets were successfully obtained in an aqueous suspension of cetyltrimethylammonium bromide (CTAB) [121]. Besides, the ethanol reduction method has also been reported to synthesize PdO/Pd-CeO_2_ hollow nanospheres [81] and core–shell Ru@AuPt nanocatalysts [211]. Methanol, as another alcohol, was also used to synthesize 3D Pd nanoparticles [212]. In addition, PtAg with nanocoral structure has been prepared through a one-step solvothermal process [137] using 1-naphthol ethanol in an autoclave at 100 °C.

#### Ascorbic Acid (AA)

Ascorbic acid (AA) is another commonly used reducing agent in the preparation of FAOR electrocatalysts. Compared to the reducing agents mentioned above, the temperature required for the AA reduction method is lower.

The practice of AA in the catalyst preparation is often accompanied by various surfactants, as shown in Fig. 10. Xu and co-workers reported the synthesis of B, P-doping PdRu nanospines for FAOR with a mass activity of 1.71 A mg_Pd_^−1^ [123], which was developed from their previous work on PdRu nanospines [110]. They used AA to reduce Na_2_PdCl_4_ at 90 °C with the assistance of KBr and Pluronic F127, followed by introducing B and P atoms through NaBH_4_ and NaH_2_PO_2_. During the reduction, the stronger coordination of Br^−^ relative to Cl^−^ caused the transformation of PdBr_4_^2−^ from PdCl_4_^2−^, thus decreasing the reduction rate [213]. Meanwhile, Pluronic, which served as the growth-directing agent, has been widely reported to prepare noble metal with branched nanostructure [214–216]. These play together to form the nanospine assemblies. SnO_2_-decorated Pd catalysts with a cubic nanostructure were also prepared by reducing Pd precursor and SnSO_4_ with AA and a surfactant hexadecyltrimethylammonium bromide (CTAB) at 95 °C [147]. N-doped graphene supported PtAu/Pt intermetallic core/dendritic shell nanocrystals were also demonstrated with the AA reduction method under the existence of sodium citrate and PVP in an oil bath [158]. This process has also been reported for the synthesis of star-like Au@Pt [217], Pd cubes [153, 218, 219] and flower-like PdAu [220] as FAOR catalysts.

AA was also applied in organic solution for synthesizing FAOR catalysts, such as PtBi@Pd hexagonal nanoplates [221] and PtSnBi nanoplates [171] with OAm and octadecene (ODE) as both solvent and stabilizer [222], PdBi nanocatalyst [223], PdZn nanocrystals [224] and PdCu nanoclusters [225] in DMF.

#### Gas Reduction

In the preparation of FAOR catalysts, various gases have been reported to be used as the reducing agents. Among all gases used, hydrogen (H_2_) is the most common reducing agent as it is clean, cheap and relatively simple to use. Besides, carbon monoxide (CO), benefiting from its ability as both the reducing and capping agent, is often applied to synthesize catalysts with nanosheet structures.

The application of hydrogen usually occurs together with high-temperature treatment, named the impregnation–reduction method. This process usually requires the use of support materials that can be immersed into the catalyst precursor suspension. This step allows precursors to be adsorbed onto the support and then is reduced under the high-temperature hydrogen atmosphere. Yang and co-workers reported the fabrication of nanoscaled Pd supported on boron-doped graphene (BG)–carbon nitride (CN) [160]. The Pd precursor was mixed with the support BG-CN followed by hydrolyzing and crystallizing at 250 °C under He/H_2_ atmosphere. Due to the support–metal particle interaction, the H_2_-reduced Pd nanoparticles showed small particle size and narrow size distribution. This led to an enhanced ECSA and high mass activity of 2.215 A mg_Pd_^−1^ toward FAOR. An ordered PdBi nanoparticles catalyst has also been reported by using the H_2_ impregnation–reduction method at 200 °C [139]. A similar strategy was also reported by Chen and co-workers to prepare structurally ordered PtCoNi ternary intermetallic electrocatalysts [226]. Other noble metals (including Ir [227] and Rh [129]) and supports, such as carbon black [228, 229], graphene [230, 231], CNT [232–234] and SiO_2_ [235], were also explored for the impregnation–reduction method.

In some studies, hydrogen can be omitted but only rely on high temperatures. Tayler and co-workers reported the preparation of PtIr nanoparticles for FAOR by using block copolymer templates. In this work, polystyrene-block-poly(4-vinylpyridine) (PS-b-P4VP) micelles were first self-assembled into a thin film as the template [236]. Pt and Ir precursors were then absorbed into the thin film, followed by thermal annealing under the argon atmosphere to reduce metal precursors and remove templates. Benefiting from the pyridinium-rich domains in the PS-b-P4VP, the prepared nanoparticles were confined to these domains, thus leading to the optimized particle distribution and controlled particle size (~ 4–13 nm).

Carbon monoxide (CO) is another common reducing agent. Compared with the H_2_ reduction where the crystal obtained is in non-directional growth with spherical morphology, CO is not only a strong reducing agent, but a surface confining agent (soft template) because CO can strongly adsorb on the Pd {111} facet and consequently confine the growth of Pd along the < 111 > direction [237, 238]. As a consequence, the catalysts prepared by the CO reduction method usually show non-spherical shapes, such as nanowires and nanosheets. Zhang and co-workers reported perforated Pd nanosheets prepared by the CO reduction method. Metal precursor Na_2_PdCl_4_ was first dissolved into methanol. Argon gas was then introduced into the solution to remove the dissolved oxygen, followed by purging CO to reduce the precursor [109]. Benefiting from the surface confining effect of CO, the formed Pd catalyst showed a nanosheet structure with a thickness of only 1.5 nm. Based on this work, researchers from the same group further investigated the influence of the surfactant on the CO-reduced Pd nanosheets [170]. They found that the introduction of PVP did not change the morphology of Pd nanosheets. However, the remained PVP was challenging to be completely removed even after being washed with ethanol and deionized water. This study concluded that strongly attached PVP suppressed the formic acid adsorption and electron transfer, suggesting the advantage of the surfactant-free preparation process [239, 240].

In order to control the morphology of nanoscaled Pd catalysts to tune their performance toward FAOR, Pramanick and co-workers compared the fabricated Pd catalysts by using various reduction gases [105]. It was demonstrated that metal precursors reduced by hydrazine, H_2_ and CO with the assistance of CTAB finally formed nanoparticles, nanowires and nanosheets with a hexagonal structure, respectively.

#### Other Reducing Agents

In addition to the commonly used reducing agents discussed above, some other reducing agents and methods have also been reported and achieved excellent results.

The thermal decomposition of M(CO)_6_ (M = W or Mo) produces metal and CO, which are both served as the structure-directing agents for the preparation of a nanosheet structure. For example, nanoflowers assembled by Pd/PdWCr nanosheets were formed in a DMF-based mixture with Na_2_PdCl_4_, Cr(CH_3_COO)_3_, W(CO)_6_ and AA, following by reduction under high temperature [119]. The results showed that W(CO)_6_ played an important role in forming the layered structure of nanosheets, and only aggregated nanocrystals were obtained without W(CO)_6_. Similarly, ultrathin PdCu nanosheets were synthesized by reducing metal precursors Pd(acac)_2_ and Cu(acac)_2_ into an oil bath at 60 °C with Mo(CO)_6_ as the reductant [108]. An ultrathin PdIrCu catalyst with a nanosheet-constructed flower was also prepared for FAOR through the mixture of metal precursors, AA and W(CO)_6_ in DMF [241].

The application of formaldehyde (HCHO) for the preparation of FAOR catalysts has also been reported and is often accompanied by the hydrothermal process and surfactants. As shown in Fig. 10, formaldehyde has been demonstrated selectively binding to some special crystal facets, such as Pd (111), thus promoting the directional crystal growth and the evolution of polyhedral structure [242, 243]. Qiu and co-workers reported the preparation of porous Pd nanosheets that were knitted by numerous interweaved ultrathin nanowires [117]. The preparation was conducted with the hydrothermal process where the pH value of the precursor solution played a determined role in the structure of the formed catalysts. Chen and co-workers published a route to prepare core–shell CuPd@Pd tetrahedra with concave structures [77]. A porous PtAg nanocatalyst with an octahedral structure was also obtained by using HCHO as the reducing agent and polyallylamine hydrochloride (PAH) as the surfactant [87]. A similar method was also used to prepare PtPdRhAg octahedral nanoframes [90], polyhedral PtPd [242] and tetrahedral PdFe [84].

The formic acid reduction method was reported from our research group for in situ growing Pt-based nanowires on the carbon paper surface to fabricate gas diffusion electrodes (GDEs) in an aqueous solution at room temperature [93, 244, 245]. Regarding the formation of the nanowires, it might be attributed to two aspects: First, the slow reduction rate at room temperature provides the opportunity for anisotropic growth [246]. The order of the crystal facet energy is (111) < (100) < (110) for Pt fcc structure, which facilitates the growth along with the closed-packed < 111 > direction following the lowest energy principle. Besides, during the formic acid reduction process, the working reducing agent is the formate anion that is produced from formic acid via the dehydration reaction. Previous studies reported that the dehydration of formic acid is favored on other Pt crystal facets compared with (111) facets. This thus assists the growth of Pt along the < 111 > direction to form the nanowire nanostructure. Pt [247], PdIr [248], PdAu [249] and PtAu [250], Pd@Pt [251] catalysts were also prepared by using the same strategy. Very recently, the fabrication of a thin and porous catalyst layer based on self-assembled jointed Pd polyhedra was reported, which is achieved by a modified formic acid reduction method. The crystal growth was modulated by using NO^3−^ to control chemical reaction balance, and the formed Pd polyhedra provided highly active jointed interfaces and high-index facets, boosting their catalytic activity toward FAOR [252].

Furthermore, citric acid [253, 254], hydrazine [255–258], sodium citrate [259–261], sodium hypophosphite [262–264], carnitine [265], benzoic acid [266] and ethanolamine [267] have been reported as the reducing agents in recent studies to prepare FAOR electrocatalysts.

#### Electrochemical Methods

Electrochemical methods, including electro-reduction/deposition and galvanic replacement, are also frequently used to synthesize FAOR electrocatalysts. The process of the electrochemical methods is usually conducted under a related eco-friendly condition, which means no surfactant, reducing agent, high temperature and pressure are required, but can provide a rapid reaction rate.

#### Electroreduction and Electrodeposition

The electro-reduction/deposition method, which uses electricity as the “reducing agent,” has been applied to synthesize electrocatalysts composed of various elements. In the application, this method is often assisted by a template to achieve designed nanostructures.

Ding and co-workers reported the preparation of mesoporous Pd nanotube arrays using a dual-template-assisted electrodeposition method. Aluminum anodic oxide (AAO) serving as the hard template was first deposited onto an Au layer, followed by adding phytantriol as the soft template [95]. The prepared dual template was placed in an electrolytic cell, and electrodeposited Pd. A group of experiments were set up in this work to demonstrate the effect of the competitive relationship between radial dendrite growth and axial growth on the formation of nanotube structures. The presence of the soft phytantriol template generated a strong electrostatic field near the AAO wall, which eliminated the radial dendrite sprout to form the thin nanotube wall. Besides, the crystal growth during the electrodeposition was randomly perturbed by the soft template, thus leading to the formation of lattice defects (such as grain boundary and twin) and step edges for improving the catalytic activity [268]. Another template-assisted electrodeposition process was demonstrated by the same research group [118]. In this study, phytantriol was aged, and self-assembled into the lyotropic liquid–crystal (LLC) phase serving as the liquid–crystal template. Pd precursor was then electrodeposited onto the template with different temperatures and potentials. With optimized conditions, the palladium membranes with periodically ordered mesopores could be obtained after removing LLC in ethanol. Nanoporous PtCuAu thin film with an ultralow Pt loading was obtained through a two-step electrochemical method [269]. The formation of the continuous nanoporous structure was obtained by the co-electrodeposition of Pt, Au and Cu, followed by a dealloying step for selective Cu removal. A dendritic Pt-Cu_2_O nanocatalyst was prepared by sequentially electrodeposited Pt, and the dendritic-shaped nano-Cu_2_O was obtained through a dynamic hydrogen bubble template (DHBT) technique onto a glass carbon (GC) surface [270]. Furthermore, the synthesis of PtBi [271], PtPd [272] and Ru@Pd [273] through the electrodeposition method was also reported.

#### Galvanic Replacement

The galvanic replacement uses the difference in the standard reduction potential between two metals to create a redox pair, in which the metal with a high reduction potential acts as the oxidant and another element works as the reducing agent (template). This template, or called the sacrificial phase, is often first formed through the chemical reaction method mentioned above, followed by the galvanic replacement reaction to prepare the designed catalyst. Various nanostructures of catalysts can be tuned by using different driving forces of galvanic replacement, which could be controlled by adjusting the ratio of the template to the metal precursor [274]. Among all elements, Ag [275–277] and Cu [278, 279] are the most common sacrificial phases used for replacement.

A bimetallic PdCu homogeneous alloy catalyst with a multipod structure was obtained through the galvanic replacement between Cu seeds and Pd^2+^, and simultaneous reduction of Cu ions to metallic Cu by oleylamine (OAm) reduction [136]. Using CuO_2_ as the sacrificial template, the preparation of Pt nanoparticles supported on CeO_2_ nanoboxes was reported by the sequential galvanic replacement of Cu^+^ with Ce and Pt in two subsequent steps [168]. A similar process was also applied to form PdCuCo catalysts for the FAOR based on two replacements (Co to CuCo to PdCuCo) followed by electrochemical dealloying [280]. Chen and co-workers synthesized PdNi hollow nanocrystals with a dendritic shell through galvanic replacement [79]. The Ni nanoparticles were first obtained by the NaBH_4_ reduction method. Then, unlike conventional replacement that is usually conducted under the protective gases, e.g., N_2_ and Ar to avoid oxidation, oxygen was introduced into the galvanic replacement process in this study. The researchers noted that the presence of oxygen promoted the surface oxidation of the Ni template to form NiO, leading to dendritic nanostructures. Besides, more bi- and tri-metal catalysts were prepared by using this method, such as PdAg hollow catalysts [91], PdFe [281] and PtNi nanoparticles [282], as well as PdAg@Pd core–shell nanotubes [106], Pt-PdFe [283] and PdCuFe nanoparticles [284].

#### Effects and Removal of Surfactants

**Fig. 11:**
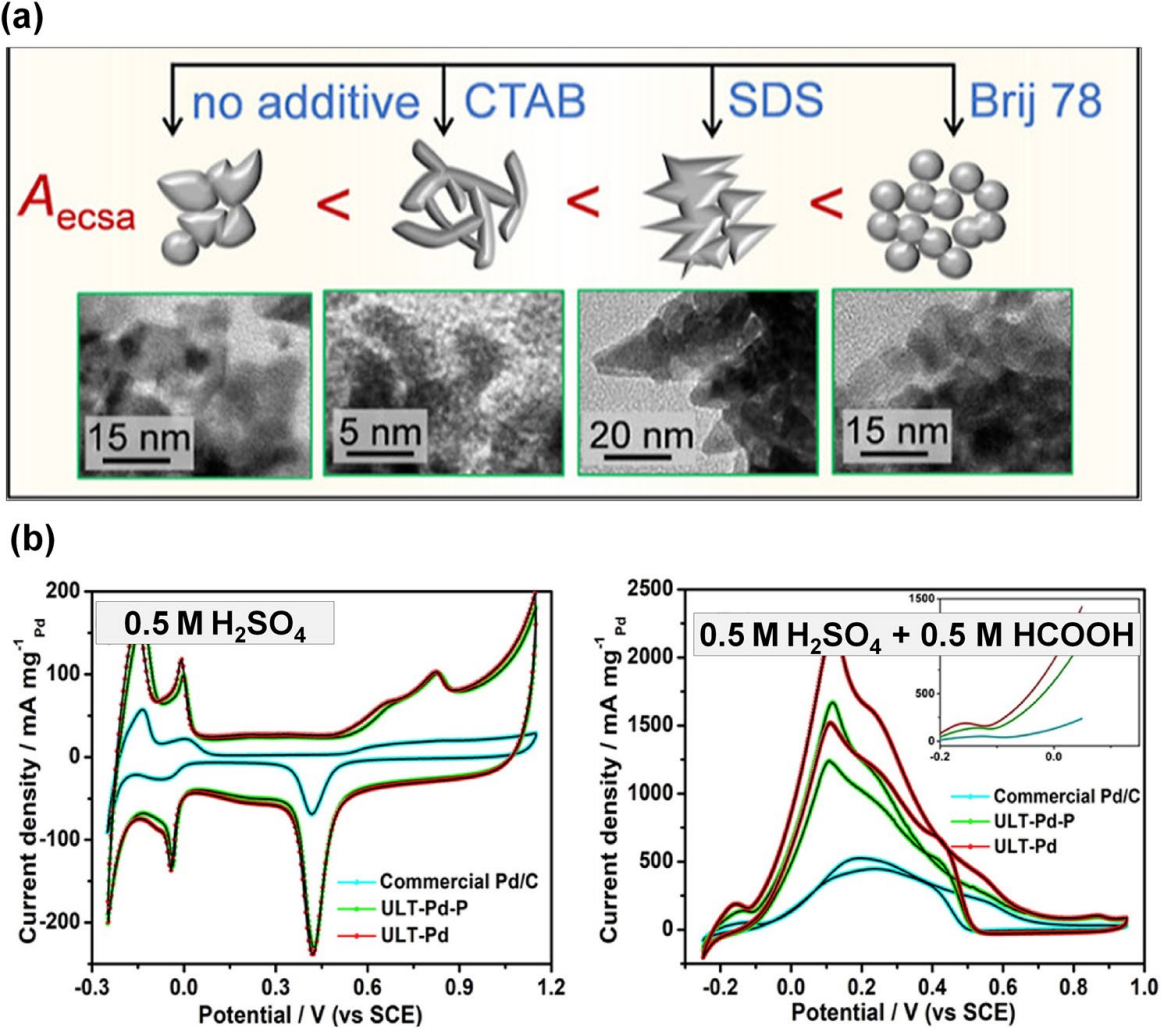
The effect of surfactants. **a** Influence of surfactants to nanostructures and electrochemical surface area (ECSA) [288]. **b** Comparison of CV curves for the Pd catalysts synthesized with/without PVP modification [170]

Based on the various preparation methods discussed above, it is obvious that organic reagents have been widely applied for different purposes. For example, surfactants or capping agents, such as PVP and CTAB, could restrict the excessive growth or induce the directional growth of nanocatalysts, as demonstrated in Fig. 11a. Besides, oleylamine (OAm) can serve not only as the reducing agent, but as the solvent in other methods. However, the removal of organic reagents is a big challenge as they are strongly adsorbed on the catalyst surface. Among all reviewed studies, few researchers note the influence of these chemicals on the activity of the formed catalysts. A good example was demonstrated in a recent study conducted by Zhang and co-workers [170]. In their work, a comparison study was conducted to the Pd catalysts formed by the CO reduction method with PVP (UTL-Pd-P) and without PVP (UTL-Pd) as the surfactant. With PVP, the catalyst showed a similar ECSA, but its peak current density for the FAOR is only 74.3% of that without PVP (Fig. 11b). The remained PVP on the catalyst surface suppressed the adsorption of formic acid as well as the electron transfer process.

The conventional process to remove organic reagents is heat treatment, while this method has many limitations as a high temperature usually leads to agglomeration and shape transformation, especially for extreme small nanoparticles or those ultrathin structures (such as nanowires, nanosheets) [285, 286]. Besides, a few attempts with other methods were reported, including UV-light cleaning [287] and chemical washing [108]. These studies show great potential, but systematic investigations are required to explore efficiency and expand their applications. Therefore, the breakthroughs are necessary to develop the universal process to remove organic reagents but maintain the structure and properties of the formed nanocatalysts.

## Meter Scale: Electrode, DFAFC and Stack

The catalysts with high catalytic activity and stability could be integrated into the DFAFC, which is a pivotal transition from theoretical catalyst design to tangible real-world applications. This process involves several disciplines, such as the fabrication of catalyst electrodes and membrane electrode assemblies (MEA), optimization of mass transfer and system management. This section will discuss the recent studies about the DFAFC electrode and single cell, and explore the catalyst-device gap.

### Configuration of DFAFC

**Fig. 12:**
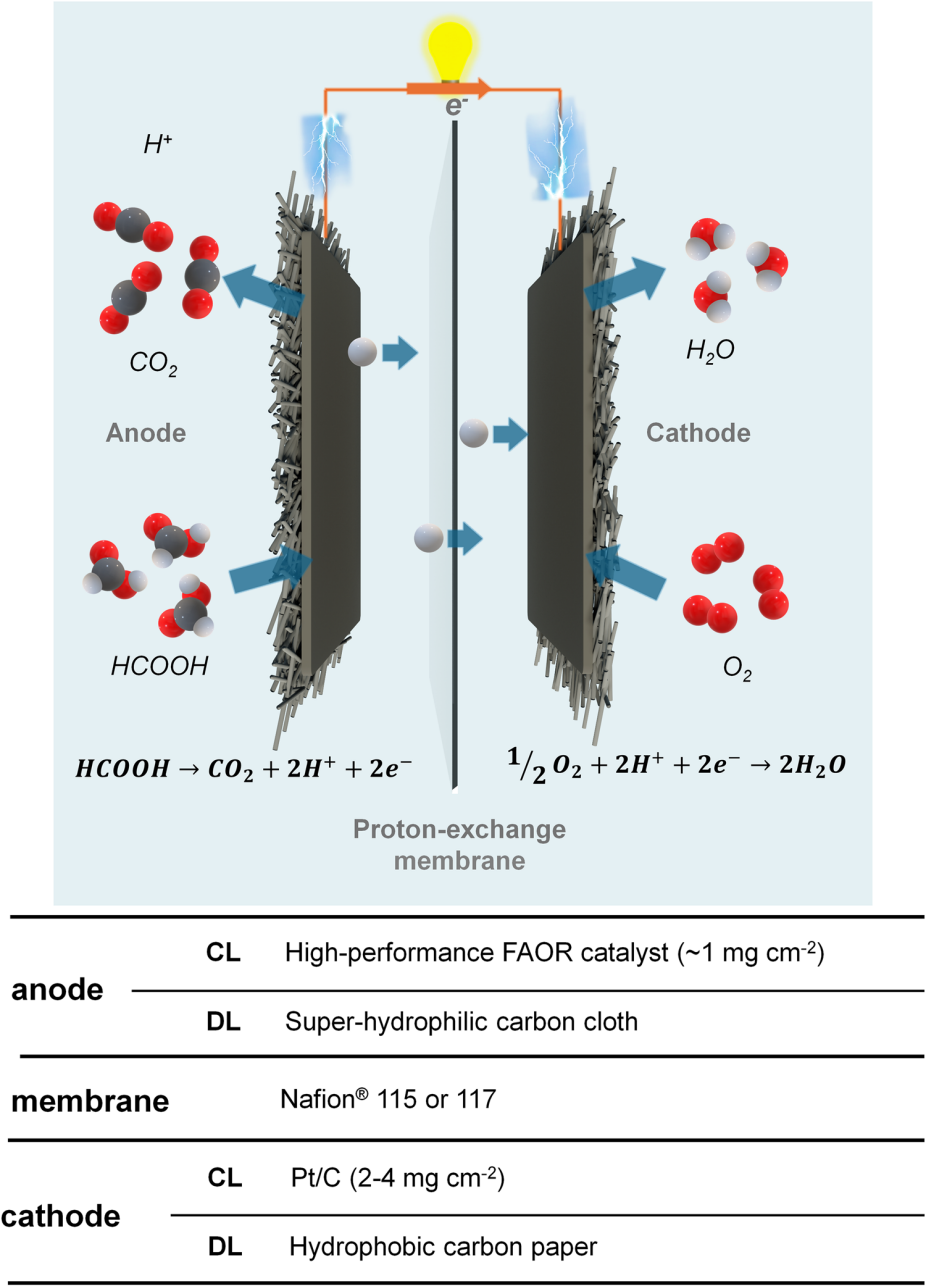
General operating principle of a direct formic acid fuel cell (DFAFC) and the present state of the anode/cathode (catalyst layer (CL) and diffusion layer (DL)) and membrane

The configuration structure of a DFAFC is similar to a hydrogen-PEMFC, as shown in Fig. 12. The primary components include a membrane in the middle and two electrodes at both sides, which is named as the membrane electrode assembly (MEA). The electrochemical reactions occur at the two electrodes. Formic acid is fed at one electrode (anode), and catalytically oxidized into protons, electrons and carbon dioxide. Protons diffuse through the membrane while electrons travel through the external circuit and reach the other electrode (cathode). Meanwhile, an oxidant, such as oxygen or in the form of air, is fed into the cathode, where the oxygen reduction reaction (ORR) is completed and water is produced as a by-product. Both simultaneous reactions lead to the electrical current through the external circuit, thus providing power.

As mentioned, the catalyst electrodes in a DFAFC include an anode and cathode where FAOR reaction and ORR happen, respectively. The latter has received the most attention because it is also a sluggish reaction and a big challenge in the hydrogen-PEMFC, and a number of excellent review papers have well-discussed catalysts/electrodes for ORR from different aspects [289–292]. In the DFAFC, the slow reaction of FAOR in the anode plays a decisive role in the fuel cell performance. Therefore, the research and development of DFAFC electrodes pay attention to the pursuit of the high-performance anode from the very beginning.

### Electrode and MEA Fabrication

Designing catalysts with excellent activity for FAOR is the foundation for the DFAFC, while the electrode and MEA fabrication process play the essential role for making a high-performance device. Unlike the thin catalyst film used in the rotate disk electrode (RDE) electrochemical measurement with the liquid electrolyte where the results are highly determined by the intrinsic catalytic activity of catalysts, the performance of a catalyst electrode is influenced by many factors. For example, an electrode usually requires a much higher catalyst loading amount, a few milligrams per square centimeter compared to the microgram level in the RDE measurement; thus, the catalyst layer in the electrode is much thicker. This results in a poor catalyst utilization, so the mass transport behavior must be considered during the practical operation [293].

Mass transport behavior in the fuel cell is primarily influenced by the gas diffusion layer. Unfortunately, to our knowledge, a gas diffusion layer specifically developed for DFAFCs has yet to be reported. However, we can refer to research on mass transport in hydrogen-PEMFCs. Currently, gas diffusion layers used in fuel cells are mainly carbon spheres supported by fibers, with the catalyst loaded onto the surface. A wide range of commercial products is already available, covering various pore sizes, thicknesses and conductivity. Meanwhile, research is ongoing to further optimize the balance between reaction kinetics and mass transport [294]. For example, Kim and co-workers reported a novel inverse opal structure [295]. This ordered microporous diffusion layer features open and interconnected pore architecture, thus showing a good effective porosity. During the fuel cell test, the fabricated electrode demonstrated effective mass transfer, and satisfactory water management, while the concentration loss was minimized. The (interfacial) resistance is another important factor that mainly depends on the materials and assembly process. Numerical calculations indicate that replacing traditional carbon materials with metals can result in lower ohmic resistance [296]. However, its corrosion issues, particularly in acidic environments, need to be addressed.

**Fig. 13:**
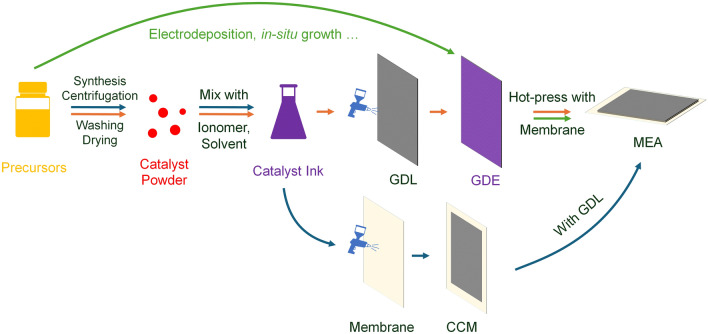
Schematic for the electrodes and MEA fabrication

After selecting a suitable diffusion layer, the next step is fabrication of DFAFC electrodes and MEAs, in which several approaches have been developed, as shown in Fig. 13. Among them, the catalyst-coated substrate (CCS) method based on the direct spraying technique is the most common technique for fabricating MEAs. With this approach, the catalyst is first prepared by different methods, such as wet chemical reduction or impregnation–reduction method. (Details have been discussed in the above section.) A separation step is required in most studies after that, including centrifugation, sedimentation and drying, to obtain dry catalyst powder. The formed catalyst is then mixed with proton conducting ionomer solution in organic solvent, e.g., isopropyl alcohol (IPA), to prepare catalyst ink, followed by being painted or sprayed onto a carbon paper GDL surface, and finally hot-pressed with a cathode and membrane to build a MEA [297]. However, this painting/spraying-CCS method suffers from the complex process of depositing catalyst powder onto the substrate. Many steps, such as centrifugation, drying and sonication, have also a big impact on catalysts prepared by changing their nanostructure, promoting agglomeration and causing low catalyst utilization. To optimize the MEA fabrication process, more facile approaches have been developed. Electrodeposition, due to its advantages of fast preparation of self-supported electrodes without binder, received great attention [298]. By adjusting electroplating parameters (such as potential and deposition time), catalyst particles with controllable size and distribution were directly deposited onto a carbon fiber paper and used as the anode [299]. Moreover, the in situ growth methods, such as formic acid reduction and hydrothermal process, have been reported to fabricate electrodes for hydrogen-PEMFCs [245] and electrolyzers [300], and can reduce the metal precursors directly onto the substrate. Very recently reported studies also demonstrated an implementation of the in situ growth method for the fabrication of DFAFC electrodes [244, 252].

Another approach, catalyst-coated membrane (CCM), was considered as the 2nd generation fuel cell fabrication technique, and has been commonly applied in PEMFC manufacture. Several attempts of CCM in DFAFCs were also reported in recent research [152, 193, 301]. As shown in Fig. 13, instead of painting/spraying the catalyst ink onto a GDL, the catalyst ink was directly painted/sprayed onto a polymer electrolyte membrane surface, leading to close contact between the catalyst layer and the membrane. Compared with the CCS method for fabricating MEAs, the CCM fabrication method is considered that has great advantages in reducing catalyst loadings, and optimizing the efficiency, power density as well as ohmic/charge transfer resistance [302]. However, due to the lack of deep investigation in optimizing the electrode structure and assembly process for DFAFC application, the power density values reported did not significantly differ from the performance of CCS-DFAFCs, as shown in Table 1. This could result from the much high catalyst loading amount compared to the hydrogen fuel cell; thus, it requires better catalyst dispersion on the substrate; otherwise, this can lead to many negative effects, for example, suppressing the benefits of CCM and blocking the mass transport within the electrode. The catalyst dispersion is highly relative to the depositing process (such as drop, paint and spray), but, unfortunately, very limited studies have been published for optimizing the DFAFC performance from this aspect [303].

**Table 1:** Performance comparison of the DFAFCs reported in recent studies

Node catalyst	Anode loading /mg cm^−2^	Fabrication method	Testing temperature / °C	Cathode gas	Peak power density / mW cm^−2^	References
PtPbBi/@PtBi	0.5	CCS	80	O_2_	161	[307]
m-PtTe nanotrepang	0.5	CCS	80	O_2_	171	[308]
Jointed Pd polyhedral	1	CCS	60	Air	202	[252]
Atomic Pt clusters	0.005	CCS	80	O_2_	145	[309]
NPG-PdCuAu	0.01	CCS	80	O_2_	93.2	[310]
Pd@Pt	2	CCS	25	O_2_	146.2	[251]
PtBiPbNiCo hexagonal nanoplates	0.5	CCS	80	O_2_	321.2	[304]
PtCu NW	2	CCS	75	Air	116.3	[244]
Pd/nanoporous Au	0.015	CCS	80	O_2_	85.4	[311]
PdFe	0.5	CCM	70	O_2_	160	[297]
PdFe	1.2	CCM	65	Air	137	[301]
Pd/TiO_2_	0.5	CCM	30	O_2_	255	[312]
Pd/MWCNT	0.52	CCS	30	O_2_	112.32	[313]
PtCu/carbon capsule	2.4	CCS	80	O_2_	121	[314]
PtZn/carbon shell	1.8	CCS	80	O_2_	107	[315]
Bi-Pt	1.2	CCS	60	Air	191	[23]
Bi-PtAu	3	CCS	60	Air	135	[180]
Pt	2	CCS	70	O_2_	42	[298]
PdBi	1.2	CCM	65	Air	20	[193]
Pd/MWCNT	0.52	CCS	30	O_2_	61.88	[316]
Pd-CNNF-G	0.5	CCM	60	O_2_	35	[257]
Pd-CoP	0.3	CCM	30	O_2_	150	[152]
Pd-B/C	1.2	CCM	30	O_2_	316	[305]
Pd/C	2	CCS	60	O_2_	91	[317]
Pd/Fu-TiO_2_-C	0.5	CCS	25	O_2_	40	[318]
PdAuIr/C-Sb_2_O_5_·SnO_2_	1	CCS	100	O_2_	94	[319]
NPG-PtBi	0.02	CCS	40	Air	80	[320]
NPG-Pt	0.013	CCM	40	Air	61	[321]

### State-of-the-Art of DFAFCs

**Fig. 14:**
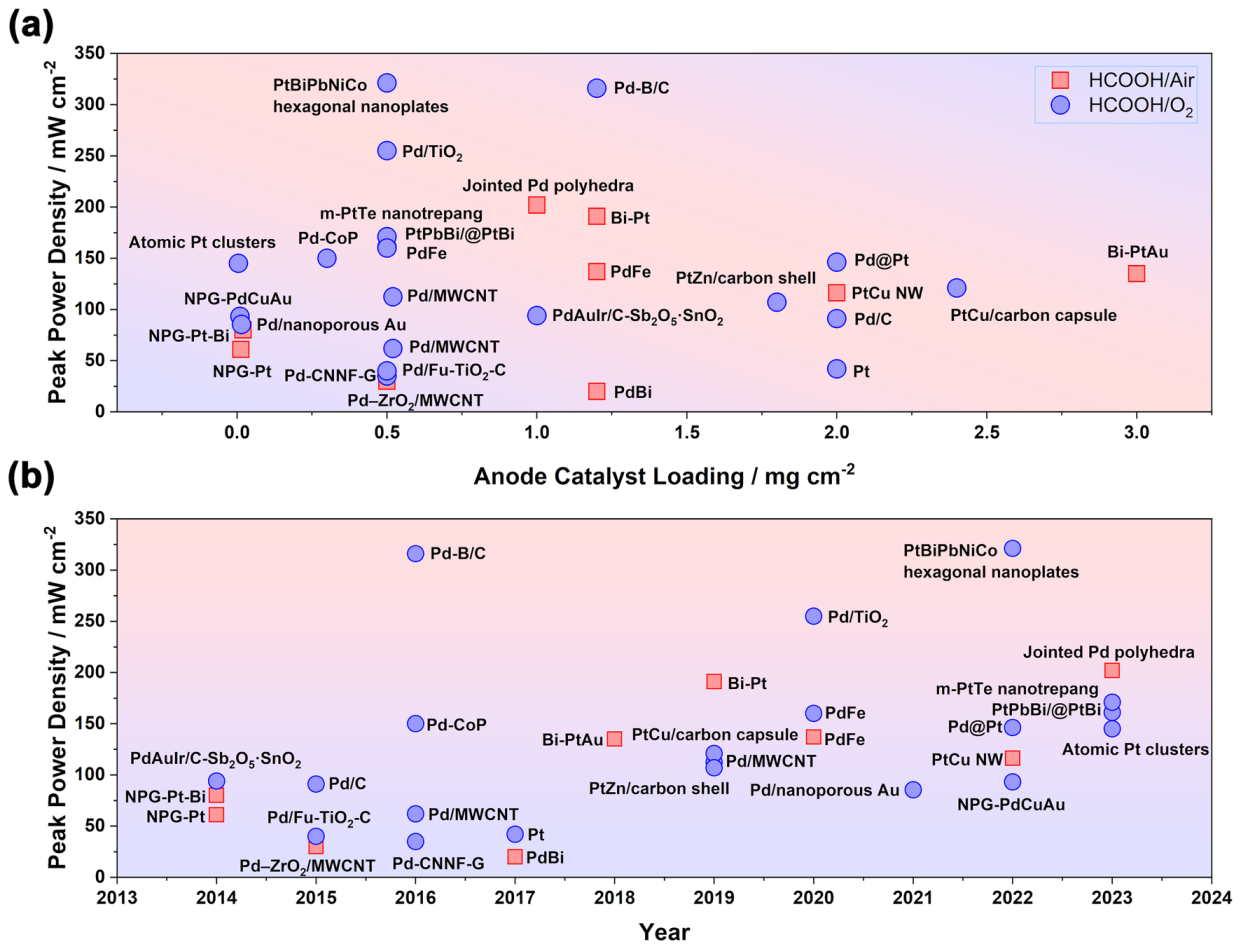
Performance of the recently reported DFAFCs. **a** Comparison of the MEAs with different catalysts at the anodes. **b** Development with time in the recently reported DFAFCs (solid cube representing the tests under air, and circle for the tests under oxygen) in terms of their peak power densities. References are listed in Table 1

Figure 14 presents the peak power densities of the DFAFCs reported in recently published studies (detailed data listed in Table 1), alongside with the catalyst loading with their respective anodes. A variety of catalysts, with differing compositions, structures and loadings, have been studied. It has been over two decades since the inception of the first DFAFC introduced in 2002. And, only in the past decade, more than 800 works have been reported on enhancing FAOR, asserting that their results could further the progress of DFAFC. However, as illustrated in Fig. 14 neither the power output nor the usage of precious metals in DFAFC seems to have experienced significant optimization in the last ten years. The peak power density in the MEA test for single DFAFCs today normally stands at below 200 mW cm^−2^, under working temperatures of 60–80 °C. However, there is significant variability in this value, with one reported at 316 mW cm^−2^ reported in 2016 and one at 321 mW cm^−2^ in 2022 [304, 305]. To the best of our knowledge, the highest value recorded to date has reached 550 mW cm^−2^ [306]. This variation can be attributed to the absence of a standard testing procedure, resulting in diverse testing conditions at present.

The common anode catalyst loading is around 0.5–2 mg cm^−2^, although instances with ultralow loadings reported at 10 μg cm^−2^ for PdCuAu and 15 μg cm^−2^ for PdAu. The diffusion layer at the anode side typically employed hydrophilic carbon cloth to ensure adequate mass transport, owing to the usage of diluted formic acid (typically 3 M) as fuel which possesses unsatisfactory permeability. In terms of membrane, Nafion® 115 and 117 are commonly used. Despite thick membranes compromising proton conductivity, they ensure reduced fuel crossover and robust mechanical strength, especially in the acidic organic solvent environments as in the DFAFC. The configuration of the cathode is similar to that of the hydrogen-PEMFC, adopting Pt/C electrocatalysts on hydrophobic carbon paper, but it requires a relatively higher catalyst loading (usually 2–4 mg cm^−2^) in the DFAFC. The majority of studies still employed pure oxygen at the cathode to overcome the reaction barrier of the sluggish ORR. It can be seen that, even a high catalyst loading (4 mg cm^−2^) was usually used at the cathode side to ensure a better kinetic activity toward ORR, the power density values recorded in HCOOH/Air are still slightly lower compared to those using HCOOH/O_2_. This indicates that besides FAOR as the rate-determining step, the mass transport improvement at the cathode side might also be another approach to further improve the power performance of DFAFCs.

**Fig. 15:**
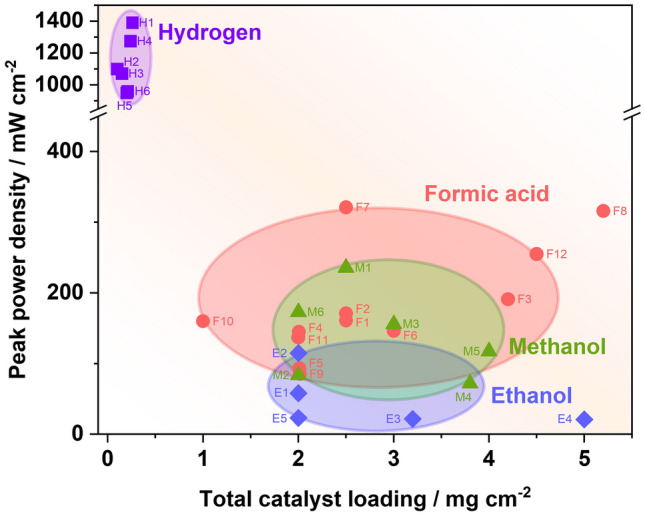
Comparison of the peak power density and total catalyst loading of different types of fuel cells. References: **Hydrogen**: H1[324], H2[325], H3[326], H4[327], H5[328], H6[329]; **Methanol**: M1[330], M2[331], M3[332], M4[333], M5[334], M6[335]; **Ethanol**: E1[336], E2[337], E3[338], E4[339], E5[340]; **Formic acid**: F1[307], F2[308], F3[23], F4[309], F5[310], F6[251], F7[304], F8[305], F9[311], F10[297], F11[301], F12[312]

In recent studies of liquid fuel cells, DMFCs, direct ethanol fuel cells (DEFCs) and DFAFCs constitute over 80% of the literature [322, 323]. Figure 15 compares their performance and catalyst usage, along with hydrogen-PEMFCs. It is evident that all three types of liquid fuel cells require relatively high catalyst loadings due to the sluggish oxidation of organic molecules. Among them, performance of the reported DMFCs is slightly lower than the DFAFCs, largely due to significant methanol crossover. The C–C bond in ethanol makes complete oxidation challenging, resulting in the currently low performance of DEFCs. Additionally, compared to hydrogen-PEMFCs, all liquid fuel cells are disadvantaged in both performance and catalyst usage. The next section offers several proposals for further optimizing DFAFCs.

**Fig. 16:**
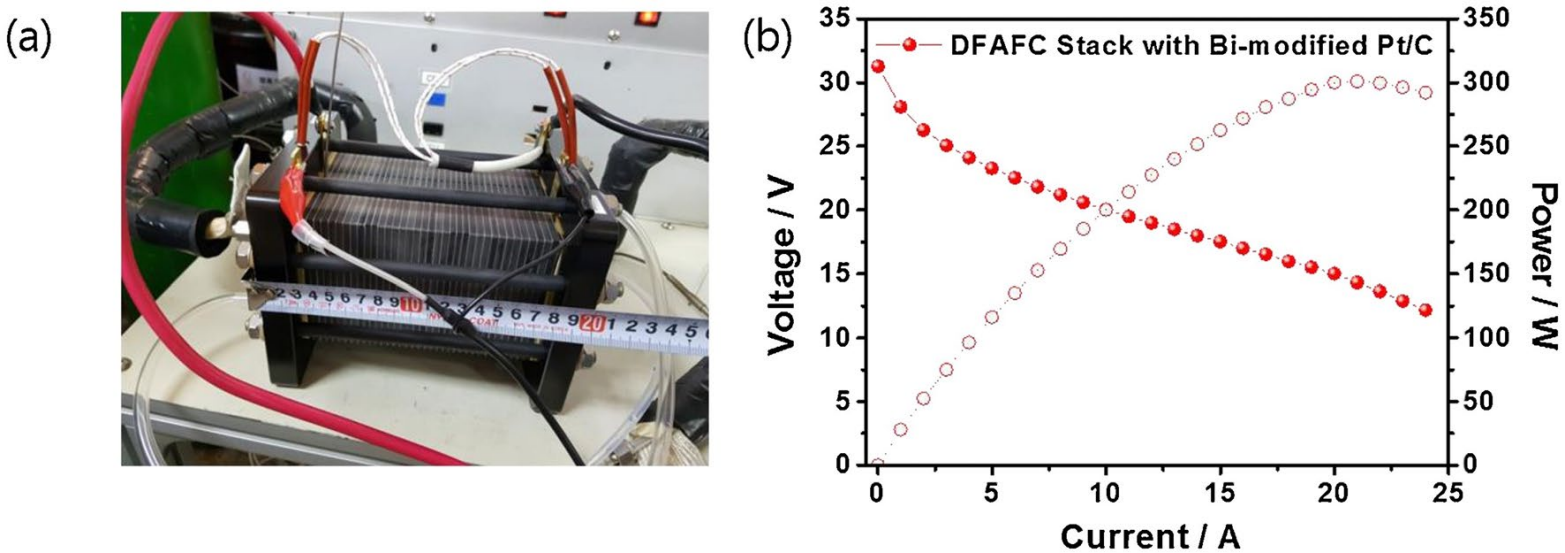
DFAFC stack. **a** Detailed photo. **b** Performance curve [23]

A fuel cell stack comprises numerous single MEAs, and is a crucial component for the commercialization of fuel cells. Unfortunately, as DFAFC research is still in its early stages, no commercial products have been reported to our knowledge; only a limited number of laboratory-scale stacks have been documented. A research paper published in 2006 reported a hybrid power source based on a DFAFC stack for a laptop computer. The stack was comprised of 15 single cells, weighing 650 g and delivered a power output of 21 W [39]. A similar class DFAFC stack was demonstrated in 2012, where the total active area was optimized to 500 cm^2^ (10 MEAs with a total noble metal of 2 g). Its stability was evaluated by a 10-day continuous lifetime test at room temperature [341]. A particularly notable study was published in 2018, in which a stack was composed of 35 MEAs, with an active area of 50 cm^2^ (Fig. 16). The stack was about 1.77 L (156 × 116 × 98 mm^3^), and contained 2.1 g Bi-Pt and 5.25 g Pt black. A maximum power density output of 300 W was recorded at 60 °C when supplying 6 M formic acid (50 mL min^−1^) at the anode and humidified air (5 L min^−1^) at the cathode [23].

### Catalyst-Device Gap

**Fig. 17:**
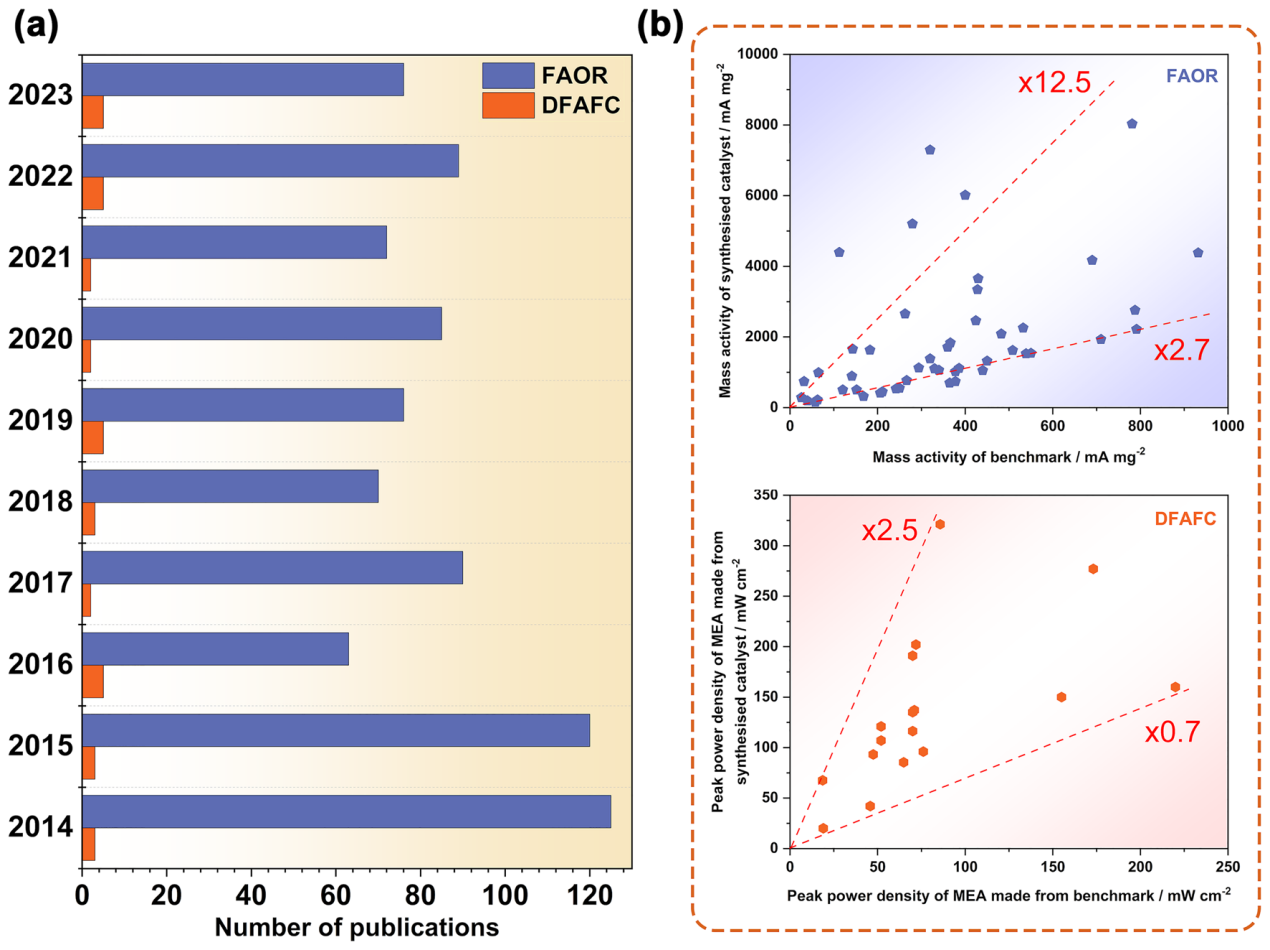
The gap between formic acid oxidation (FAOR) catalysts and real tests in direct formic acid fuel cells (DFAFCs). **a** Number of the research studies reported. **b** Comparison of the enhancement reported for the activity of the catalysts measured in the half-cell measurement in the liquid electrolyte (FAOR) and power density of the practical electrodes in the single-cell test (DFAFC), showing as a ratio of the reported catalysts to the benchmark catalysts

Electrooxidation of formic acid is the most crucial process in the DFAFC. Thus, most of efforts have been focused on the improvement of the catalytic activity of anodic catalysts. In the studies reported, most of the formed electrocatalysts demonstrated excellent catalytic performance toward FAOR in the electrochemical measurement, and their authors predicted the prepared catalysts would show great performance in the application of DFAFCs. A literature survey about the research in this area (Fig. 17a) shows more than 60 papers were published annually within the last decade, proposing new strategies to boost FAOR, such as preparation of novel nanostructures, alloy and supports; however, less than 5% reports really made their catalysts to electrodes and tested their performance in the MEA in fuel cells. As a consequence, no significant progress has been reported and the challenges of high catalyst loading and low power density faced by the DFAFC technology is still not addressed.

The gap between the development of catalysts and electrodes is also reflected in the comparison of the performance improvement of the reported catalysts to commercial catalysts (Fig. 17b). Most studies use the thin film RDE measurement in liquid electrolytes to evaluate the intrinsic activity of their prepared catalysts. Through the calculation of average mass activity from recently reported catalysts, a 2.7- to 12.5-folder improvement value was achieved compared to the commercial catalysts for the FAOR catalytic activity by the RDE measurement. In some studies, extremely high mass activity was recorded, which was even hundreds of times higher than that of benchmarks used in their report [127, 129, 133]. On the other hand, the improvement of the power density of single fuel cells reported in the MEA test is much smaller. Only a 0.7- to 2.5-folder improvement ratio was obtained when using the as-prepared catalysts in the anode electrodes. As compared with the two graphs in Fig. 17b, catalysts tested in the MEAs all demonstrated much lower enhancement compared with their mass activity recorded in the RDE measurement. This big difference between the RDE measurement and MEA test for the enhancement monitoring is mainly caused by the more complex conditions of the catalysts in practical electrodes during the fuel cell operation, so the results obtained by the RDE measurement in the liquid electrolyte cannot accurately reflect their behavior in a single cell [74]. In the electrochemical measurement represented by RDE, the catalyst loading required is ultralow (usually ~ 20 µg cm^−2^) [342]; thus, only a thin film catalyst layer is formed. However, applying catalysts to electrodes usually need a 100-folder higher catalyst loading in DFAFC electrodes. In addition to possible agglomeration, as mentioned above, this also leads to a very thick catalyst layer (can reach 50 µm at 2 mg cm^−2^), resulting in an extremely high mass transport resistance [343]. Besides, a thick catalyst layer tends to impede the uniform distribution of the ionomer and fails to provide a substantial number of triple phase boundaries (TPBs) that are essential for electrochemical reactions. These factors all cause a dramatic decrease in catalytic utilization, and suppression of transport of both formic acid fuel and the produced CO_2_ during the fuel cell operation.

Based on the aforementioned considerations, we propose that a high-performing DFAFC should exhibit the following characteristics:

*High-performance catalysts:* An ideal catalyst for DFAFCs should demonstrate superior electrocatalytic activity specifically for FAOR, ensuring rapid and efficient fuel conversion. Beyond its activity, the catalyst must be selective, targeting the desired reaction pathway and minimizing side reactions. Its performance should remain stable over prolonged exposure to formic acid and high temperature, resisting any form of degradation or poisoning. A significant number of achievements have been made in this aspect as discussed in last section. Moreover, the catalyst layer needs to be thin, optimizing the distribution of ionomer, thus offering more TPBs.

*Electrode structure with optimized mass transport:* The diffusion of reactants and products in a DFAFC is pivotal for its performance, requiring an optimized porosity for effective fuel transport and swift removal of products like carbon dioxide. The hydrophobicity of this layer needs to strike a balance, promoting gas repelling while facilitating necessary mass transport. In the context of durability, the total electrode structure should exhibit mechanical stability, resisting severe degradation over time. Some early studies about the mass transfer have been reported, including temperature [344], pores in the catalyst layer [345], diffusion media structure [346] and flow channel [347]. However, more comprehensive and in-depth investigations are expected to predict the optimal structure based on the practical operation requirement in application in the future.

*Acid-resistant low-crossover membrane:* Membranes stand, as a crucial component in DFAFCs, serves as the separator between the anode and the cathode, ensuring that the fuel on the anode side does not directly mix with the oxygen on the cathode side, while simultaneously allowing the passage of ions to maintain electrical neutrality. Given the corrosive nature of the formic acid, its material should inherently resist chemical degradation. Even under an environment of a high formic acid concentration, the membrane should retain its mechanical integrity and resist dissolution, swelling or even rupture. Currently reported studies about DFAFC, in order to maintain mechanical strength and minimize the fuel crossover, typically employ thick membranes, such as Nafion® 117 (183 µm). However, this approach substantially increases proton conduction resistance. Consequently, an ideal membrane should be as thin as possible. Based on our knowledge, there is no study specifically aiming at optimizing membranes for DFAFCs that has been published to date. This review, therefore, does not delve into the issue of the membrane.

## Perspective

**Fig. 18:**
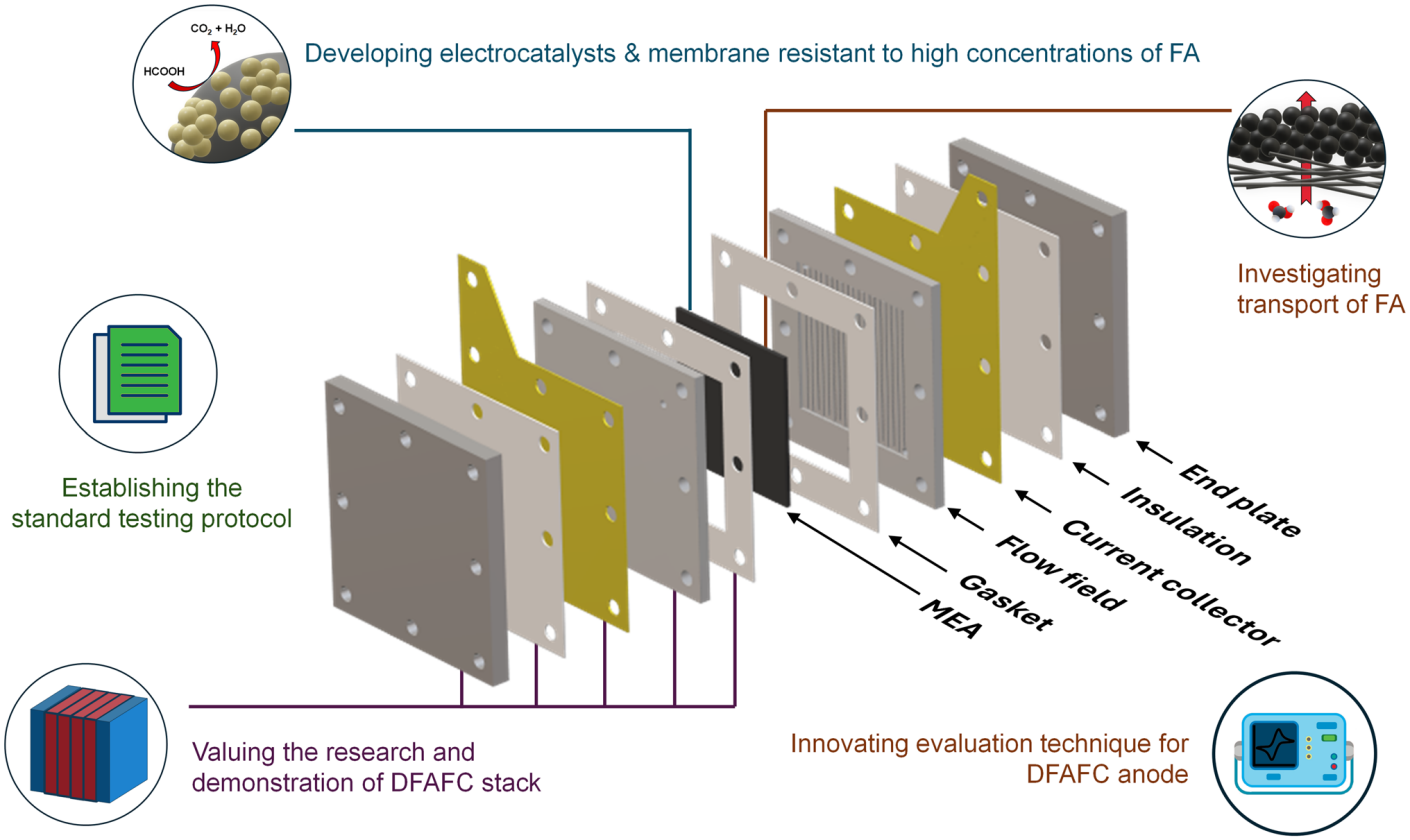
Schematic illustration represents the perspectives of the future development of the direct formic acid fuel cell (DFAFC)

To further develop DFAFC and be used as an efficient power source in the industry, several challenges cannot be overlooked, as shown in Fig. 18.

### Low Power Output

One of the major challenges with a DFAFC is the relatively low power energy output. Formic acid has a high-energy density, which stands out in comparison with other potential fuels and serves as one of the foundational motivations for pursuing the DFAFC technology. This high-energy density theoretically suggests that DFAFCs could achieve impressive high power outputs. However, during practical demonstrations, the power outputs of DFAFCs typically only reached about 200 mW cm^−2^. For comparison, hydrogen-PEMFC can achieve more than 1 W cm^−2^ [348]. One of the critical reasons for this discrepancy is that the current DFAFC can only utilize diluted formic acid, implying that the majority of fuel pumped into the anode is water, leading to low fuel energy density. For example, DFAFCs typically use 3 M formic acid, which is diluted, reducing the energy density from 1770 to around 200 Wh L^−1^ (Fig. 1). This is significantly lower than the energy density of other fuels, such as approximately 1500 Wh L^−1^ for 700 bar H_2_, rendering DFAFCs less competitive.

The impact of high concentrations of formic acid on catalysts is not yet fully understood. One of the primary concerns with concentrated formic acid is catalyst poisoning. Most of the development and electrochemical measurements reported on catalysts for DFAFCs have been conducted using low concentrations of formic acid. This means that the understanding of the behavior, efficiency and longevity of these catalysts in the presence of concentrated formic acid is still in its infancy. High concentrations of formic acid might also potentially alter the dominant reaction pathways; thus, there is a heightened risk of undesirable side reactions that produce species which can adsorb onto the catalyst surface and block its active sites. Furthermore, it is unclear whether concentrated formic acid can lead to rapid physical degradation of the catalyst. With a higher fuel concentration, the rate of reactions at the catalyst surface might increase. While this could potentially lead to higher immediate power outputs, it might also result in accelerated wear and tear of the catalyst over time, reducing its lifespan. The corrosive nature of formic acid can also erode the substrate and the binder materials, leading to a loss of catalyst material or reduced surface area available for the reactions. This requires further studies to answer these questions.

Another significant factor preventing the use of more concentrated formic acid in DFAFCs is the membrane. Its performance and stability are vital for the efficient operation of the fuel cell. However, when exposed to high concentrations of formic acid, the membrane encounters fatal issues. Formic acid, as a strong organic solvent with corrosive properties, can interact with the polymer matrix of the membrane, leading to accelerated chemical degradation, especially at elevated temperatures. This interaction can lead to the dissolution and swelling of the membrane material, and even cleavage of polymer chains. This can alter the membrane's ionic conductivity, increase its permeability and lead to a reduction in its mechanical strength. Over time, these adverse effects can culminate in the thinning or the formation of pinholes in the membrane, ultimately leading to breaches [349]. Excessive fuel crossover can result in the FAOR happening at the cathode, which not only wastes fuel but also degrades the cathode's performance over time. A more severe compromise in the membrane integrity could lead to direct contact between the anode and cathode, resulting in a short-circuiting of the entire DFAFC system. Thus, using diluted formic acid becomes a pragmatic approach. It mitigates the direct exposure of the membrane to high concentrations of formic acid, thus extending its lifespan and maintaining the overall efficiency of the DFAFC. Therefore, it is necessary to develop more resilient and chemically stable membrane materials that can withstand higher concentrations of formic acid, potentially unlocking greater efficiencies and power densities for DFAFC.

In addition to using highly concentrated formic acid, another potential approach is to use concentrated formate salts, such as HCOOK and HCOONa. This could effectively avoid the issues associated with highly concentrated acid. Some studies have already utilized diluted formate salts as a direct fuel supply for DFAFCs [350]. However, formate fuels typically require the addition of KOH [95], creating an alkaline environment, which presents a greater challenge. On the one hand, current research indicates that the peak current for FAOR is lower in alkaline conditions [61], necessitating the development of tailored electrocatalysts. On the other hand, the PEM must be replaced with an anion exchange membrane (AEM) due to the lack of protons, yet most AEMs are still at the experimental stage and lack sufficient durability. Consequently, this approach faces a long development pathway.

### Limited Catalyst Loading

Structural engineering shows a significant effect on the catalytic performance of a catalyst through controlling the nanostructures during the synthesis. As shown in Fig. 6, the activity of catalysts toward FAOR is critically affected by their ECSA. However, the studies by the RDE measurement usually use a very low catalyst loading to maintain good dispersion and prevent aggregation to obtain intrinsic activities. This will become a challenge when incorporating such catalysts to fabricate electrodes. The fabrication of electrodes needs significant quantities of catalysts using intricate procedures. In such scenarios, the benefits of the superior catalysts and the issue of their retention within the practical electrodes become somewhat unclear. This is one of the major challenges that prevent the scale-up of the catalysts with novel structures to achieve a high-performance electrode, thus resulting in the commonly known catalyst-device gap. In hydrogen-PEMFCs, some progress has been achieved to bridge this gap between the highly active electrocatalysts and the poor performance device, including both the catalyst evaluation technique, such as with the half-cell test using the floating electrode technique and the gas diffusion electrode test approaches [244, 351], and the distribution of proton conducting ionomer on the catalyst interface by nitrogen-doping catalyst support, catalyst surface modification and hydrophobic feature control of the catalyst layer structure [352]. These techniques might also be explored for their applications in the development of novel electrocatalysts for DFAFCs. Besides, direct growth methods show some possibility for addressing this gap issue, but fine control over the structure of the fabricated catalyst layer can still not meet the practical application requirements. This demands further investigations in both the process control and the fundamental understanding to really understand the formed catalyst structure-performance relationship.

### Diffusion Layer and Mass Transfer

For fuel cells operated at a high current density region, the efficiency of mass transport determines the performance of DFAFCs. Without adequate fuel supply to the catalyst layer, even the most exceptional catalyst would be rendered ineffective. It is widely known that the challenge of oxygen mass transfer resistance in the cathode of PEMFCs is complex, comprising bulk/molecular, Knudsen and catalyst/ionomer interfacial transport resistances [98]. However, the mass transport in DFAFCs might be even more complicated. This needs consideration not only on the diffusion of formic acid molecules, but also the expulsion of the produced carbon dioxide. Contrary to alcohols, which can readily permeate hydrophobic diffusion layers even at low concentrations, formic acid exhibits poor permeability at low concentrations, thus heavily relies on superhydrophilic diffusion layers. Despite some studies have initiated discussions on the fuel transport and bubble distribution [345, 346], research focused on diffusion layers for formic acid remains in its nascent stages. Recent advancements in numerical simulation techniques [347], especially in the development of machine learning-based optimization, offer promising avenues for designing optimized transfer channels, highlighting the significant potential for breakthroughs in this domain.

### Development of DFAFC Stack

Stacking is an indispensable step toward the commercialization of DFAFCs, while it has not received enough attention. Whereas this can be ascribed to the numerous challenges that individual MEAs still face, system-level research often aligns more closely with commercial applications. Advancements in this domain can attract increased attention, which in turn can spur research into individual MEAs and various subsystems. A prime example of this is the DMFC. Despite the many challenges associated with using methanol as a fuel, there are still some instances of its successful commercialization. DMFC stacks have been employed in various areas, encouraging researchers and industries to invest more efforts into the study of different components, such as the development of catalysts and membranes. This virtuous cycle offers a valuable pathway for the progression of DFAFCs.

### Establishment of a Standard Testing Protocol

The current testing conditions for DFAFCs are highly diverse, leading to a wide variation in the power outputs obtained. This makes it challenging to compare the results from different studies. There is an urgent requirement to establish a standard testing protocol. For instance, in the case of hydrogen-PEMFCs, the DoE (Department of Energy, USA) has released the standard “Procedure for Performing PEM Single-Cell Testing.” This not only lays a foundation for evaluating the performance of different MEAs, but also aids in setting further research targets. Such a move can provide researchers worldwide with a clear research direction, subsequently enabling targeted optimization of DFAFCs.

## Conclusions

Formic acid has been demonstrated to be a new class of safety fuel for the fuel cell, attributed to its properties of easy storage and transport, high cell potential and low crossover. The research on formic acid over the past century has provided insights into the mechanism of FAO. Dual parallel pathways, as a widely accepted mechanism, prove the presence of adsorbed CO as an intermediate during the oxidation process. This is considered the main cause of catalyst poisoning. With the help of modern in situ/operando and simulation technologies, more intermediates have been identified, among which formate is considered an important intermediate. The adsorption of its two forms (monodentate and bidentate) was found can regulate the reaction rate, while related research is gradually improving. Based on the understating of the mechanism, many studies were reported to propose strategies to improve the catalytic activity toward FAOR, and develop new synthesis routes to obtain designed catalysts. Significant progress has been achieved from three main aspects. (i) Optimizing electronic structure through the preparation of alloys; (ii) obtaining high surface area through the design of nanostructures; and (iii) enhancing stability through the application of supports. Benefiting from these achievements, formic acid was proposed for use in fuel cells, and made great progress, including the development of high-performance catalysts and successful demonstration of the DFAFC stack. However, to promote the commercialization and be used as an industrial-scale fuel cell, several challenges in different scales cannot be overlooked; in particular, the power output of DFAFC is relatively low, as it restricts its wider application. Exploring the use of higher concentration formic acid fuel emerges as a potential strategy. This approach places more demanding requirements on catalyst development to meet the increased challenges of catalyst poisoning and stability.
